# Selection of *In Vivo* Predictive Dissolution Media Using Drug Substance and Physiological Properties

**DOI:** 10.1208/s12248-020-0417-8

**Published:** 2020-01-27

**Authors:** Deanna M. Mudie, Nasim Samiei, Derrick J. Marshall, Gregory E. Amidon, Christel A.S. Bergström

**Affiliations:** 1 Global Research and Development, Lonza, Bend, Oregon 97703 USA; 2 Department of Pharmacy, Uppsala Biomedical Centre, Uppsala University, P.O. Box 580, SE-751 23 Uppsala, Sweden; 3 Present Address: Pivotal Drug Product Technologies, Amgen, Cambridge, Massachusetts 02141 USA; 4 College of Pharmacy, Department of Pharmaceutical Sciences, University of Michigan, Ann Arbor, Michigan 48103 USA

**Keywords:** bicarbonate, biorelevant, buffer, dissolution, solubility

## Abstract

The rate and extent of drug dissolution in the gastrointestinal (GI) tract are highly dependent upon drug physicochemical properties and GI fluid properties. Biorelevant dissolution media (BDM), which aim to facilitate *in vitro* prediction of *in vivo* dissolution performance, have evolved with our understanding of GI physiology. However, BDM with a variety of properties and compositions are available, making the choice of dissolution medium challenging. In this tutorial, we describe a simple and quantitative methodology for selecting practical, yet physiologically relevant BDM representative of fasted humans for evaluating dissolution of immediate release formulations. Specifically, this methodology describes selection of pH, buffer species, and concentration and evaluates the importance of including bile salts and phospholipids in the BDM based upon drug substance log *D*, p*K*_a_, and intrinsic solubility. The methodology is based upon a mechanistic understanding of how three main factors affect dissolution, including (1) drug ionization at gastrointestinal pH, (2) alteration of surface pH by charged drug species, and (3) drug solubilization in mixed lipidic aggregates comprising bile salts and phospholipids. Assessment of this methodology through testing and comparison with literature reports showed that the recommendations correctly identified when a biorelevant buffer capacity or the addition of bile salts and phospholipids to the medium would appreciably change the drug dissolution profile. This methodology can enable informed decisions about when a time, complexity, and/or cost-saving buffer is expected to lead to physiologically meaningful *in vitro* dissolution testing, *versus* when a more complex buffer would be required.

**Electronic supplementary material:**

The online version of this article (10.1208/s12248-020-0417-8) contains supplementary material, which is available to authorized users.

## INTRODUCTION

One of the many challenging tasks facing formulators developing and testing drug candidates is the selection of the optimal dissolution medium with which to conduct *in vitro* tests. The goal is to select a medium and test protocol that produces *in vitro* results that accurately reflect the rate and extent of drug dissolution *in vivo*—an increasingly difficult task, given the quantity of complex molecules in drug pipelines and the variety of media types and compositions available. Selection of the optimum dissolution medium depends strongly on the physicochemical properties of the drug and the fluid properties of the gastrointestinal (GI) tract.

Conventional dissolution media, such as simple US Pharmacopeia (USP) buffers (e.g., hydrochloric acid, 50-mM phosphate, acetate, and citrate) have been used for solubility and dissolution assessment for decades and are referenced in the majority of USP monographs (1,2). These media can be valuable and provide simple, reasonably accurate assessments of *in vivo* solubility and dissolution rate in some cases, such as for highly soluble, highly permeable compounds (Class 1 Biopharmaceutics Classification System (BCS) compounds) (3,4). However, these media do not mimic the properties and composition of GI fluids, which vary along the length of the intestine and exhibit high inter- and intra-subject variability (5–12) (see Table I for key GI fluid properties).

**Table I:** Relevant Properties of Fasted-State Human Gastric Fluid (FaHGF) and Human Intestinal Fluid (FaHIF) (jejunum) that Affect Dissolution

Property	Value		
	FaHGF (stomach)	FaHIF (duodenum)	FaHIF (jejunum)
pH	2.5 (median)^a^, 1.7–3.3 (range)^a^, 2.3 (median)^b^, 1.1–7.5 (range)^b^, 2.0 (median)^c^, 1.1–3.9, (range)^c^	6.3 (median)^a^, 5.6–7.0 (range)^a^, 4.9 (median)^b^, 1.7–7.6 (range)^b^	6.9 (median)^a^, 6.5–7.8 (range)^a^, 5.6 (median)^b^, 2.2–6.8 (range)^b^
Buffer capacity (mM/ΔpH)	17.9 (average)^c^, 1 to 160 (range)^c^	1.7 (average)^d^, 0.4–6.3 (range of averages)^d^	2.3 (average)^e^, 0.3–6.3 (range of averages)^e^ 2 to 13^f^
Buffer concentration (mM)/species	~ 0.5–20 mM (range)/HCl^a^	6–20 at pH 6.5/bicarbonate^g^	6–20 at pH 6.5/bicarbonate^g^
Bile salts (mM)^a^	0.28 (median), 0.0 to 0.8 (range)	3.25 (median), 2.5–5.9 (range)	2.52 (median), 1.4 to 5.5 (range)
Phospholipids (mM)^a^	0.029 (median)	0.26 (median)	0.19 (median)
Osmolality (mOsmol)^a^	202 (median), 119 to 221 (range)	197 (median), 137–224 (range)	280 (median), 200 to 300 (range)
Surface tension (mN/m)^a^	36.8 (median), 31 to 45 (range)	34–41 (range)	25–34 (range)

^a^From ref. (38)

^b^From refs. (7,8)

^c^From ref. (39)

^d^Personal communication with author of reference (7)

^e^From ref. (7)

^f^From refs. (54,55)

^g^From ref. (29)

To address the need for more accurate *in vitro/in vivo* correlations for the variety of conditions along the GI tract for poorly soluble (i.e., BCS 2 and 4) drug compounds, biorelevant dissolution media (BDM) have been developed. These BDM have evolved significantly with our knowledge of GI physiology (13–19) and include versions representative of fasted and fed states along the entire length of the GI tract (6–8,20,21). These media vary in pH, buffer species, buffer concentration, osmolality, viscosity, and surface tension, as well as the concentration and type of bile components. These media have been shown to accurately predict solubility values measured in aspirated intestinal fluid for many drug substances (22). While this diversity of available buffers and simulated media enables investigation of the dissolution sensitivity of a compound to medium composition, it can also make selection of the most practical, yet biorelevant, medium challenging for pharmaceutical scientists.

This tutorial describes a methodology for selecting the simplest and most practical BDM expected to provide physiologically relevant *in vitro* dissolution performance of immediate release (IR) formulations in the upper GI tract (stomach, duodenum, and jejunum) of fasted healthy humans. This section of the GI tract was chosen because it is often where most drug absorption occurs. Our recommendations are designed primarily to guide formulation selection and optimization by screening formulations in media comprising the key physiological parameters expected to impact dissolution. Therefore, these recommendations are suited for biorelevant dissolution testing, which typically commences during early development and may continue through clinical testing and beyond (23). However, these recommendations can also be useful for development of some quality control (QC) or clinically relevant methods. While in some cases, QC, biorelevant, and clinically relevant dissolution methods may be different, in other cases, a single dissolution method may meet the purpose and requirements of all three (23).

This tutorial complements other published decision trees in the area of *in vitro* dissolution testing and is also unique in certain aspects. For example, Andreas and coworkers have published a paper introducing the OrBiTo WP2 Decision Tree, which provides guidance for selecting *in vitro* methods for aiding oral formulation development of IR, delayed release and extended release formulations (24–28). The decision tree directs the user to different “levels” of dissolution media composition as proposed by Markopolous and coworkers (27). They present general concepts for medium selection for a range of dosage forms in the fasted and fed GI tract based upon the Developability Classification System (DCS) (26,28). This tutorial complements the general framework of Markopoulos by giving the reader tools to select a medium based on drug p*K*_a_, intrinsic solubility, and log *D* for IR dosage forms in the fasted state. While this tutorial is most applicable to poorly soluble (i.e., BCS/DCS 2 and 4) compounds, knowing BCS/DCS class is not a prerequisite. In addition, the recommendations differ from Markopolous and coworkers in the selection of buffer capacity. Markopolous defines biorelevant buffer capacity as a BDM with a buffer capacity of the bulk solution within the range of the bulk buffer capacity reported *in vivo* (i.e., for FaHIF). In contrast, we define biorelevant buffer capacity to be drug property dependent. It refers to the buffer capacity at the surface of the dissolving drug that results in surface pH and dissolution rate similar to that of physiological bicarbonate.

## DISSOLUTION MEDIUM SELECTION METHODOLOGY

### Introduction

Drug-substance and drug-product dissolution is the result of a complex interplay between dissolution medium, physiological, drug substance, formulation, and product properties. The BDM selection methodology described in this tutorial accounts for the interplay between the most important properties impacting *in vivo* performance. The methodology is based upon a mechanistic understanding of how three main factors affect dissolution: (1) drug ionization at the pH levels of the stomach and small intestine, (2) alteration of surface pH by charged drug species, and (3) drug solubilization in mixed lipidic aggregates composed of bile components (i.e., bile salts, phospholipids, and cholesterol). These three phenomena are a result of the interplay between BDM and drug-substance properties, as shown in Table II.

**Table II:** Effect of Interplay Between BDM and Drug-Substance Properties on Dissolution

BDM property	Drug-substance property	Effect of BDM-drug-substance interplay
pH	• p*K*_a_^a^	Extent of drug ionization across pH range of GI tract
	• Acid/base character	
Buffer capacity	• p*K*_a_	Extent of surface pH alteration by charged drug species
	• Acid/base character	
	• Intrinsic solubility	
Concentration of bile salts and phospholipids	• Log *P*^b^/log *D*^c^	Extent of drug solubilization in mixed lipidic aggregates

*pK*_*a*_, negative log of the acid dissociation constant (*K*_a_); *log P*, logarithm of the partition coefficient between octanol and water for a completely non-ionized molecule; *log D*, logarithm of the pH-adjusted partition coefficient of a molecule between octanol and water

**Fig. 1:**
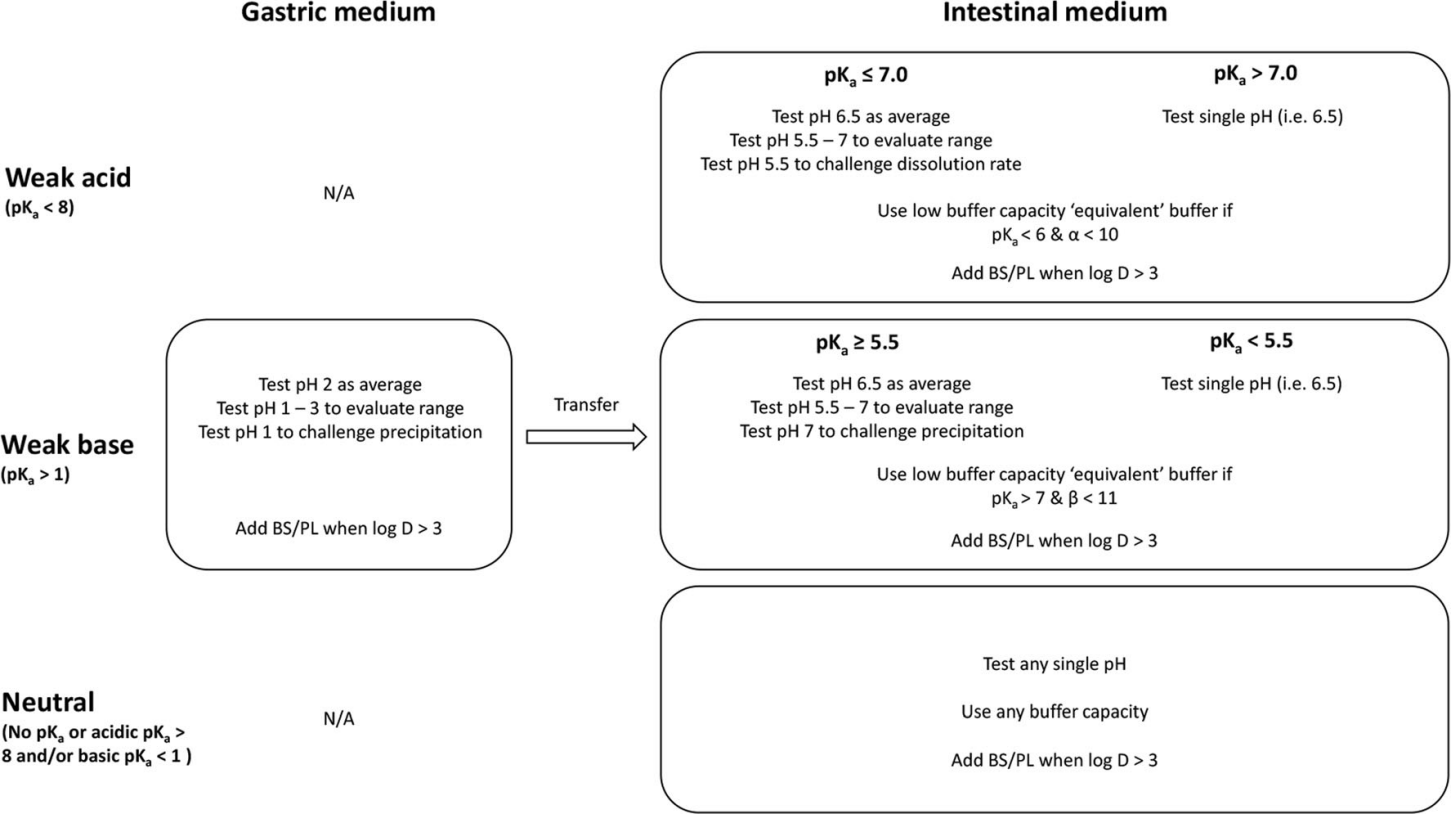
Medium types recommended for *in vivo* predictive dissolution measurements. For p*K*_a_ ≤ 7 (acids) and p*K*_a_ ≥ 5.5 (bases), three different options are provided to accommodate different dissolution testing goals. BS/PL, bile salts and phospholipids; log *D*, logarithm of the pH-adjusted partition coefficient between octanol and water. *α*, p*K*_a_-log *S*_o_; *β*, p*K*_w_-p*K*_a_-log *S*_o_. All buffers should be adjusted to an ionic strength of 0.15 M

BDM selection recommendations based upon these phenomena are summarized in Fig. 1 and described in detail in subsequent sections of this tutorial. Because dissolution can also be affected by properties such as osmolality, surface tension, viscosity, and the ionic strength of GI fluids, the recommended BDM properties were chosen to align with physiological values. While other drug-substance properties and solid-state characteristics may also affect dissolution (5,10), these properties were not the focus herein. In addition, potential impacts of excipients and dosage form design on drug release were not considered. For example, any acidic or basic excipients present in the formulation could impact the GI region/pH at which the drug is released and modulate bulk and surface pH (29–31). Therefore, recommendations are most applicable to IR dosage forms containing standard tableting excipients.

Below, we describe a methodology to select the optimum (1) pH, (2) buffer species and concentration, and (3) bile salt (BS)/phospholipid (PL) content for the BDM. We then describe confirmation of this methodology through testing and literature reviews. This evaluation showed that the recommendations correctly identified when a biorelevant buffer capacity or the addition of BS/PL to the BDM would appreciably change the dissolution profiles of the compounds. Finally, we discuss additional considerations related to *in vitro* test methods and *in silico* modeling.

### Selection of pH

This section addresses the selection of medium pH based on two main variables—drug-substance p*K*_a_ and acid/base character. Specifically, we examine how these two variables affect drug ionization at gastric and small-intestinal pH levels.

#### Theory

The rate of dissolution of a collection of drug particles in solution can be described by

1 $$ \frac{d{C}_{\mathrm{b}}(t)}{dt}=\frac{1}{V_{\mathrm{b}}}.\mathrm{Sh}.\frac{D_{\mathrm{eff}}}{R(t)}.{A}_{\mathrm{s}}(t).\left({C}_{\mathrm{s}}(t)-{C}_{\mathrm{b}}(t)\right) $$

where *C*_b_ is the measured bulk drug concentration, *t* is time, *V*_b_ is the volume of the bulk solution (i.e., the BDM), Sh is the Sherwood number (i.e., the non-dimensional flux of molecules/mass from the particle surface into the surrounding fluid, equal to the effective particle radius (*R*) divided by the diffusion-layer thickness), *D*_eff_ is the effective drug diffusivity in the dissolution medium, *A*_s_ is the surface area of the dissolving particles, and *C*_s_ is the saturated concentration (i.e., saturation solubility) at the surface of the dissolving drug particle (32). As is described below, the pH at the surface of the dissolving particle, defined as the “surface pH” influences the value of *C*_s_.

Sh in Eq. (1) accounts for potential enhancements in dissolution rate over pure diffusion, which can occur as a result of factors such as fluid shear, convection, or effects from neighboring drug molecules in a concentrated system. When dissolution occurs as a result of pure diffusion, Sh is equal to unity and effective diffusion layer thickness equals *R* (32–34).

The extent of ionization of a weak acid or weak base drug can significantly affect *C*_s_ and, therefore, the rate and extent of dissolution. The extent of ionization is dependent upon the pH of the medium at equilibrium and the drug p*K*_a_ value(s) (35,36). As pH varies between different regions of the GI tract, *C*_s_ and the rate and extent of dissolution can also vary. While weak acids tend to have low solubility in the acidic stomach and increased solubility in the small intestine, weak bases tend to have high solubility in the stomach and decreased solubility in the small intestine, where supersaturation and/or precipitation may occur (37). However, for free acids and bases, the solubility from one region to another depends upon the p*K*_a_ of the drug together with physiological factors such as pH, buffer species and concentration. The relative solubility, defined as *C*_s_/*S*_o_ can be calculated, as described in Sect. 1 and Fig. S1 of the Electronic Supplementary Materials. This value is useful for estimating the difference in solubility between the stomach and small intestine for weak acids and bases.

It is also important to consider differences in solubility within a given region due to pH variations within that region. A pH range of 1–3 brackets the median values reported in the stomach, and a pH range of 5.5–7 brackets the median pH values reported in the fasted human jejunum (see Table I) (8,38,39). Within the gastric pH range of 1–3, *C*_s_ for a weak acid is estimated to be relatively constant for drugs with a p*K*_a_ above 2.5. However, *C*_s_ is expected to vary 2-fold or greater for weak bases with p*K*_a_ ≥ 1 across this pH range. For example, for a weak base with a p*K*_a_ ≥ 4.5, *C*_s_ is calculated to vary 100-fold between pH 1 and 3. Therefore, gastric pH variation can be more impactful to solubility and dissolution rate of weak bases compared with weak acids.

As the p*K*_a_ of a weak acid decreases, the extent of ionization and therefore solubility becomes more sensitive to pH in the jejunal pH range (see Fig. S2 in the Electronic Supplementary Materials). For example, when p*K*_a_ = 5, there is a calculated 24-fold difference in *C*_s_ between pH 5.5 and 7. However, when p*K*_a_ = 7, there is only a 2-fold difference in *C*_s_ between pH 5.5 and 7. The opposite is true for weak bases. As the p*K*_a_ of a weak base increases, the extent of ionization and therefore *C*_s_ is more sensitive to pH in the range of 5.5–7. When p*K*_a_ = 8, there is a calculated 29-fold difference in *C*_s_ between pH 5.5 and 7. When p*K*_a_ = 5.5, there is only a 2-fold difference in *C*_s_ between pH 5.5 and 7.

#### Recommendations

We recommend testing weak acids in dissolution medium representative of the small intestine since a limited relative extent of dissolution is expected in the stomach. We recommend testing weak bases in a sequential gastric to intestinal dissolution medium, since a high relative extent of dissolution is expected in the stomach followed by supersaturation/precipitation in the small intestine (See Fig. 1). A “pH-dilution” method such as that performed by Gao and coworkers (40) or multicompartment methods could be employed when testing bases in a sequential dissolution transfer test, for example, from pH 2.0 to 6.5 medium (40–53). Since neutral drugs do not ionize over the intestinal pH range, they can be tested in any single pH medium.

For weak acids with p*K*_a_ ≤ 7 and weak bases with p*K*_a_ ≥ 5.5, the reader can choose to study dissolution and/or precipitation at (1) an average pH, (2) over a pH range, or (3) at either the low or high end of the range. Selection of one or multiple options may depend on whether the reader is seeking to understand performance over a range (i.e., option 2) or wants to develop a discriminating test (i.e., option 3).

A pH of 2 was selected as the average gastric pH. A range of ~ 1 to 3 has been reported for gastric pH in fasted healthy humans after they have ingested a glass of water (7,11,38). A pH of 6.5 was selected as the average intestinal pH, as it falls between the values reported in two recent publications and is the pH of *in vitro* surrogates of small-intestinal fluids (e.g., the pH of original FaSSIF and FaSSIF-V2) (13,27). We recommend both gastric and intestinal buffers to be adjusted to an ionic strength of 0.15 M using NaCl to reflect average ionic strength in the GI tract (9).

The goal of these recommendations is to minimize the number of pH values that must be tested, while still gaining information about potential variations in dissolution rate for pH-sensitive drugs. In addition, exposing an acidic or neutral drug to gastric medium could be important if the formulation comprises excipients whose disintegration or dissolution may be impacted differently in acidic compared to moderate pH medium. While the approach above is specific to monoprotic weak acids and bases, it can also be applied to drugs that have multiple p*K*_a_ values as described in Sect. 4 in the Electronic Supplementary Materials.

### Selection of Buffer Species and Concentration

This section addresses the selection of buffer species and concentration, specifically examining the effects of drug p*K*_a_, acid/base character, and intrinsic solubility (*S*_o_) on surface pH.

#### Theory

As an ionizable drug dissolves and goes into solution, it can decrease the fluid pH (acid) or increase the fluid pH (base) when the buffer capacity of the fluid is not sufficiently high. Both cases would lead to a lower percentage of drug ionization. Therefore, solubility and dissolution rate would be lower compared to a case where the buffering capacity was high enough to withstand a potential pH change caused by dissolution of a weak acid or base.

This resulting decrease in driving force for dissolution would be reflected as a lower *C*_s_ value in Eq. (1). The buffer capacity of fluids aspirated from different regions of the GI tract from human subjects has been reported to be low. Recently, Hens and coworkers reported measured buffer capacities of aspirated fasted human intestinal fluid (FaHIF) of healthy volunteers in the fasted and fed states in the range of 2 to 6 mM/ΔpH (7). Other researchers have shown bicarbonate buffer concentrations ranging from about 6 to 20 mM in the upper small intestine, with corresponding buffer capacities ranging from 2.5 to 13 mM/ΔpH (6,38,54–57). In contrast, the buffer capacities of several BDM are at the upper end of those measured *in vivo* (see Table III), with the commonly used USP SIF (50 mM phosphate, 18 mM/ΔpH) being considerably higher. These relatively high *in vitro* buffer capacities may lead to higher *in vitro* dissolution rates than expected *in vivo* (58,59).

**Table III:** Buffer Capacities and Compositions of Bile Components and Phospholipids in of Some Common *In Vitro* Biorelevant Media and USP SIF, TS

BDM property	Value						
	FaSSGF ^a,b^	FaSSIF^c^	FaSSIF-V2^d^	FaSSIF-V3^e^		Bicarbonate^f^	USP SIF, TS^g^
Buffer species	–	Phosphate	Maleate	Maleate	Phosphate	Bicarbonate	Phosphate
Buffer p*K*_a_	–	6.69^c^	6.00^e^	6.00^e^	6.69^c^	6.04	6.69
Buffer concentration (mM)	–	28.7	19.1	10.26	13.51	16.2	50
pH	1.6	6.5	6.5	6.7		6.5	6.8
Osmolarity (mOsmol/kg)	120.7	270	180	215		Not measured	113
Experimental buffer capacity (mM/ΔpH)	–	12	10	5.6		7	18.4
Bile salt(s) (mM)	0.08 (TC)	3 (TC)	3 (TC)	1.4 (TC), 1.4 (GC)		–	–
Phospho-lipid(s) (mM)	0.020 (PC)	0.75 (PC)	0.2 (PC)	0.035 (PC), 0.315 (LPC)		–	–
Sodium oleate (mM)	–	–	–	0.315		–	–
Cholesterol (mM)	–	–	–	0.2		–	–
Average surface tension (mN/m)	42.6	54.7	54.2	35.1		Not measured	Not available

*TC*, taurocholate; *GC*, glycocholate; *PC*, phosphatidylcholine (lecithin); *LPC*, lysophosphatidylcholine (lysolecithin)

^a^From ref. (19)

^b^Medium also contains 0.1 mg/mL pepsin

^c^From ref. (14)

^d^From ref. (16)

^e^From ref. (13)

^f^From ref. (59)

^g^From ref. (15)

To what extent a drug may change the fluid pH as it goes into solution depends not only on the pH and buffer capacity of the fluid, but also on the p*K*_a_ and *S*_o_ of the drug. For an acid, the lower the p*K*_a_ relative to the starting pH of the buffer and the higher its *S*_o_, the higher its propensity to lower the pH as it dissolves. For a base, the higher its p*K*_a_ relative to the starting pH of the buffer and the higher its *S*_o_, the higher its propensity to increase pH as it dissolves. In both cases, the concentration of ionized drug in solution can become high relative to the concentration of buffer and result in a pH change. The terms p*K*_a_-log *S*_o_ (designated *α*) for a weak acid and p*K*_w_-p*K*_a_-log *S*_o_ (designated β)^1^ for a weak base provide a means of determining the combined contribution of drug pK_a_ and drug S_o_ on the capacity of a drug to alter surface pH.

**Fig. 2:**
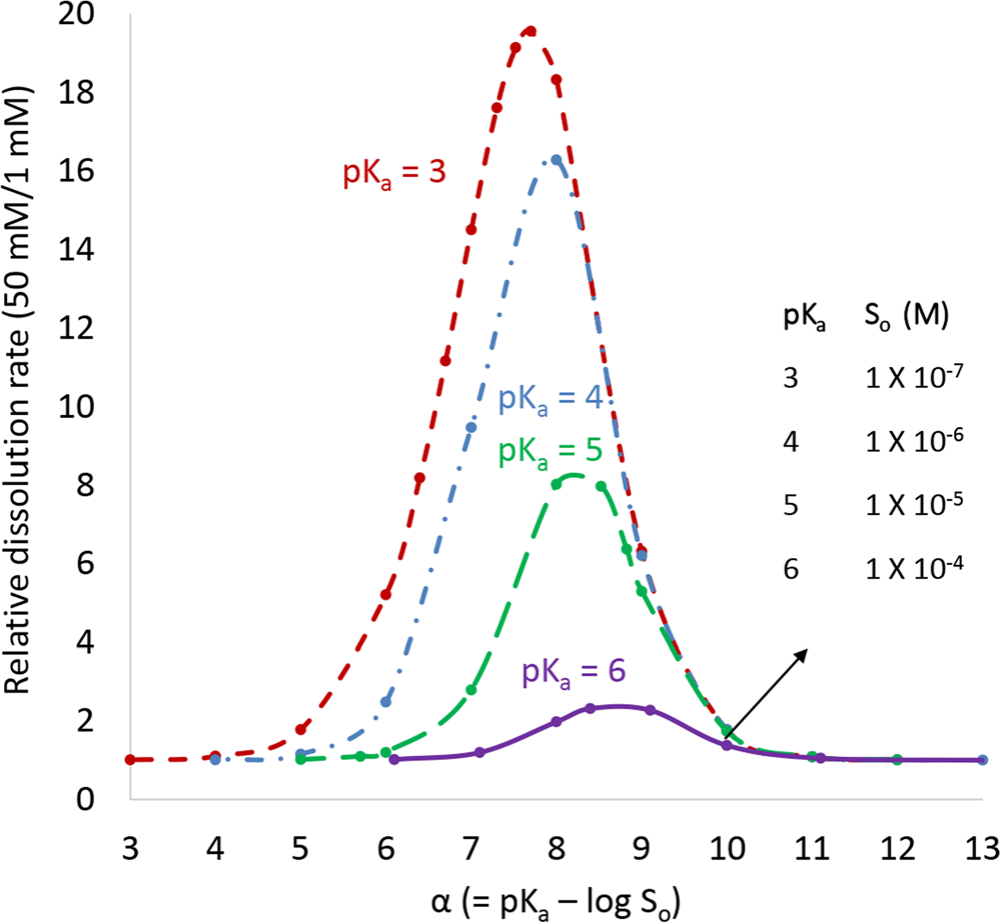
Relative predicted surface-area-normalized dissolution rate for a monoprotic weak acid in phosphate buffer at pH 6.5. Assumes different drug p*K*_a_ values at a high (50 mM) and low (1 mM) buffer concentration. Drug diffusivity = 7.9 × 10^−6^ cm^2^/s. Effective diffusion layer thickness = 30 μm

**Fig. 3:**
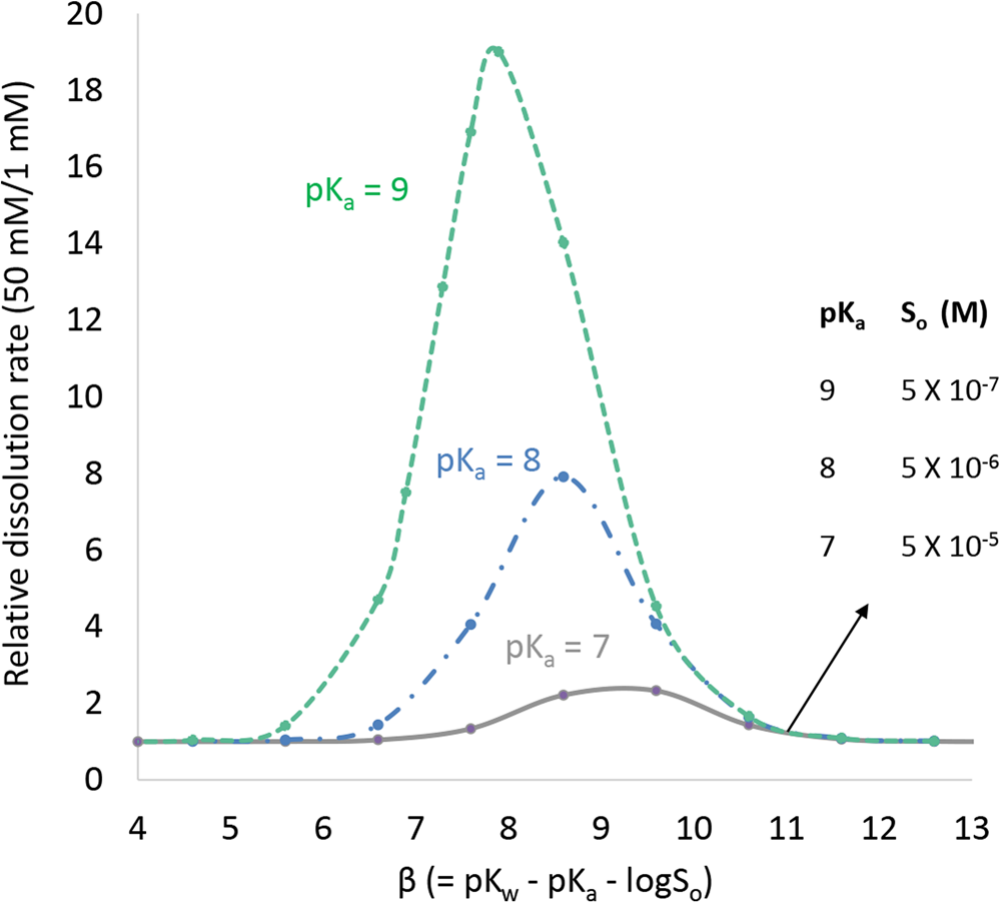
Relative predicted surface-area-normalized dissolution rate for a monoprotic weak base in phosphate buffer at pH 6.5. Assumes different drug p*K*_a_ values at a high (50 mM) and low (1 mM) buffer concentration. Drug diffusivity = 7.9 × 10^−6^ cm^2^/s. Effective diffusion layer thickness = 30 μm

Figure 2 (acids) and Fig. 3 (bases) show surface-area-normalized relative dissolution rates in a pH 6.5 phosphate buffer as a function of *α* or *β*. The relative dissolution rate is the calculated dissolution rate at high buffer concentration (50 mM) divided by the calculated dissolution rate at low buffer concentration (1 mM). When the relative dissolution rate equals unity, no differences in rate are expected over this range in buffer concentration. As shown in Fig. 2, for a monoprotic weak acid, when the drug p*K*_a_ is ≤ 6, up to a 2-fold or greater difference in dissolution rate could occur when *α* is in the range of 5 to 10. As shown in Fig. 3, for a weak base, when drug p*K*_a_ ≥ 7, up to a 2-fold or greater difference in dissolution rate could occur when *β* is in the range of 5 to 11. When *α* or *β* is outside of this range, the effect of buffer concentration on dissolution rate is insignificant. Above a value of 10 (acids) or 11 (bases), the drug *S*_o_ is low enough that the concentration of ionized drug in solution at the starting pH is too low relative to the buffering capacity to change surface pH. Therefore, the dissolution rate is near its maximum at that pH in both buffer concentrations. At low values of *α* or *β* (such as p*K*_a_ = 5 and *α* < 6), *S*_o_ is high enough that surface pH has changed to a similar extent in both buffer systems and dissolution rate is near its minimum. Section 2 in the Electronic Supplementary Materials provides plots of surface-area-normalized relative dissolution rates in pH 5.5 and 7 phosphate buffers for monoprotic weak acids and bases as a function of *α* or *β*, respectively. For a weak acid, the propensity to decrease surface pH at a given *S*_o_ is higher at pH 7 compared with pH 6.5 since pH-p*K*_a_ increases. For a weak base, the propensity to increase surface pH at a given *S*_o_ is higher at pH 5.5 compared to pH 6.5 since p*K*_a_-pH increases. Section 2 in the Electronic Supplementary Materials also provides a plot of calculated surface area normalized dissolution rate in a given buffer system relative to infinite buffer capacity. This plot can be used to estimate when a drug may have a propensity to alter surface pH over a range of buffer species, buffer concentrations, and pH values.

#### Recommendations

Based upon the impact of alteration of surface pH on dissolution, we make the recommendations for selection of buffer capacity shown in Fig. 1. If a drug substance has a propensity to alter surface pH, we recommend using buffer concentrations/capacities lower than what is achieved using a typical (i.e., USP 50-mM) buffer. The values of p*K*_a_, *α* and *β* were selected because they delineate when a greater than 2-fold difference would be expected between a 50-mM phosphate buffer concentration (i.e., USP SIF) and a 1-mM concentration with a calculated Van Slyke buffer capacity (0.5 mM/ΔpH at pH 6.5) at the lower end of the range in FaHIF. Although a difference in dissolution rate between 50 mM and 1 mM phosphate buffers is not expected at low values of *α* or *β* (for example, p*K*_a_ = 5 and *α* < 6) despite alteration of surface pH, a lower threshold is not specified since these cases would mainly be relevant for high solubility (i.e., BCS 1 or 3) drugs with *S*_o_ > 1 M.

For drugs that do not have the propensity to alter surface pH (i.e., they have values outside the range described above), any convenient buffer-capacity buffer can be used. It is unnecessary to select an “equivalent” buffer to bicarbonate, since the buffer capacity of a maleate or phosphate buffer, for example, will have little to no effect on the resulting dissolution rate, provided properties such as ionic strength are held constant. These recommendations can be applied to polyprotic drugs as described in Sect. 4 in the Electronic Supplementary Materials.

**Fig. 4:**
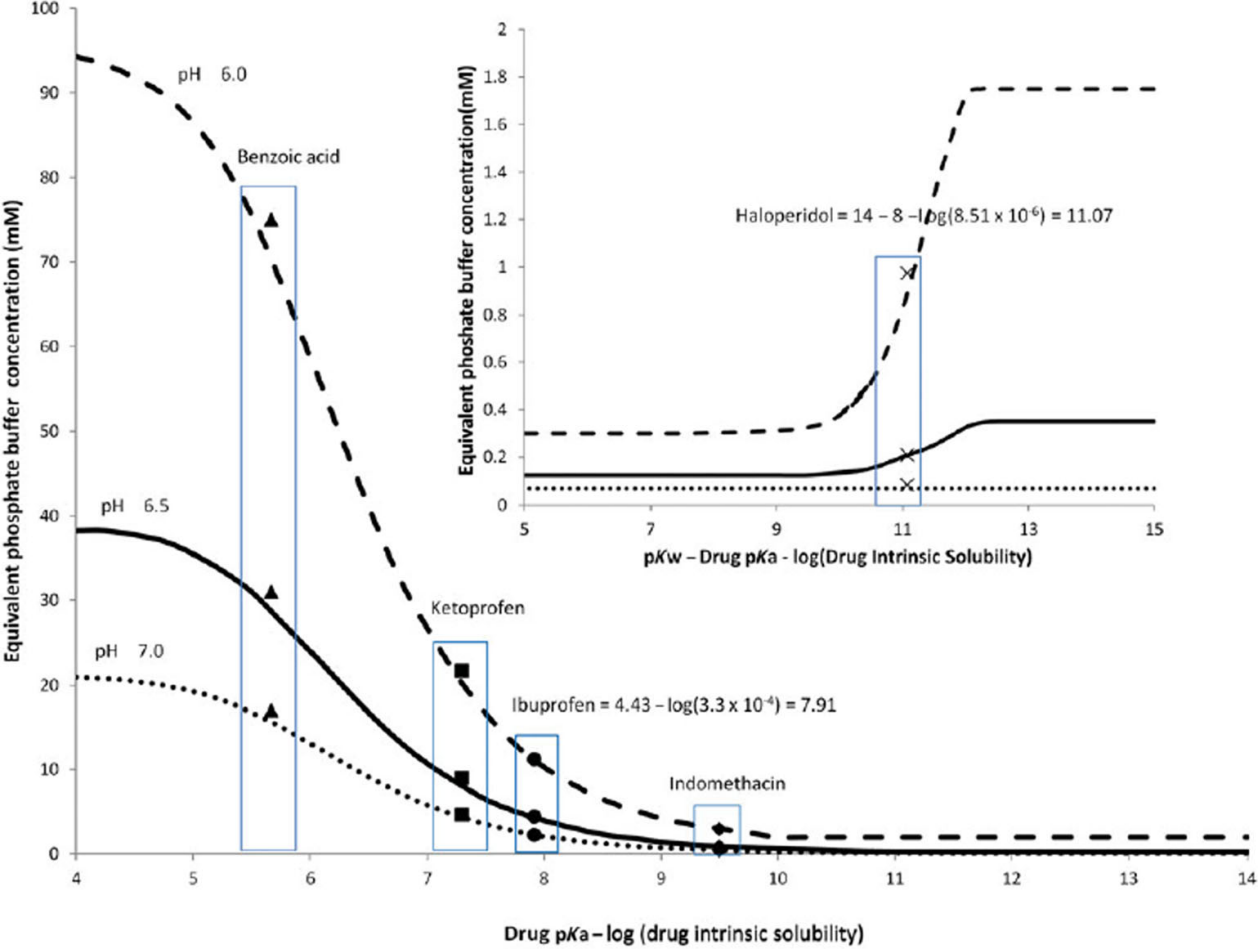
Predicted equivalent phosphate buffer concentration needed to match physiological bicarbonate buffer for weak acids and weak bases (reproduced with permission from Krieg *et al.* (59)

The method developed by Krieg and coworkers (see Fig. 4) can be used to tailor the buffer concentration of a phosphate buffer to achieve an “equivalent” buffer representative of average performance in physiological bicarbonate (48). For acids, Krieg and coworkers recommend phosphate concentrations in the range of 1 to 25 mM, depending on the value of *α*. For bases, they recommend extremely low buffer concentrations (< 2 mM) to match physiological bicarbonate buffer (59). To study the range of the expected variation in dissolution rate as a function of buffer capacity, the concentration could be expanded above and below the calculated “equivalent concentration” rather than simply relying on an average bicarbonate buffer capacity as suggested by Krieg. Because the recommendations by Krieg and coworkers are based upon experiments performed under sink conditions, it may be necessary to use a titrant to maintain bulk pH if desired when drug-substance solubility and the dose-to-volume ratio in the dissolution experiment are high (59). Since a pH of 5.5 is greater than 1 pH unit below that of phosphate, equivalent maleate concentrations for monoprotic weak acids and bases at pH 5.5 are provided in Sect. 3 in the Electronic Supplementary Materials.

The analysis described in this tutorial represents a static situation in which a drug is dissolving in a buffer within a well-mixed, closed container. In contrast, the *in vivo* situation is dynamic. In the intestinal lumen, the fluid composition and resulting buffer capacity at a given location is impacted by factors such as secretion of bicarbonate and digestive enzymes, fluid absorption, fluid transit and hydrodynamics (58). In addition, the concentration of dissolved drug is affected by absorption into the intestinal membrane or transit down the GI tract. While closed container *in vitro* dissolution devices cannot mimic this situation, using a pH-stat or multicompartmental devices that incorporate both transit and secretion move closer to capturing the dynamic situation *in vivo* (40–53).

When considering dissolution in the intestine, selecting an equivalent buffer is likely more important for weak acids than for weak bases. Whereas weak acids tend to dissolve to a much greater extent in the small intestine, weak bases tend to first dissolve in the stomach and then potentially supersaturate and or/precipitate in the small intestine. Therefore, the implication of buffer capacity on dissolution rate for bases in the small intestine is less important, particularly for small precipitates, which may re-dissolve in the intestine. In this case, the effect of bulk buffer capacity on the extent of supersaturation/precipitation/re-dissolution of bases is likely of greater importance.

While this tutorial focuses on the impact of surface pH on intestinal dissolution, surface pH in the stomach can be important, particularly for weak bases. Pepin and coworkers demonstrated the impact of surface pH on the solubility and dissolution rate of the weak base, acalabrutinib (61). They demonstrated that surface pH increased to 4.0 at a bulk pH of 2 in hydrochloric acid. Inputting surface solubility calculated from surface pH in an *in silico* model provided better predictions of experimental dissolution rate compared to overpredictions arising from using bulk solubility. These authors provide a methodology for predicting surface pH of monoprotic and diprotic weak bases (61).

### Selection of Bile Salts and Phospholipids

This section addresses the inclusion or exclusion of BS and PL in the BDM and their concentrations, focusing on the effect of drug-substance lipophilicity on solubilization in mixed lipidic aggregates.

#### Theory

Solubilization of lipophilic drug substances in mixed lipidic aggregates has the potential to increase *C*_s_ and, therefore, the rate and extent of dissolution. Intestinal mixed lipidic aggregates are mixed micelles or vesicles composed of BS, PL, and cholesterol. The aggregates present in aspirated FaHIF have been found to vary significantly between individuals and prandial state in terms of composition, size, and form (62). Riethorst and coworkers provide recent updates on the human inter-subject variability of the composition (20). In simulated intestinal media, mixed lipidic aggregates can be present as vesicles or swollen micelles with approximate diameters of 45 nm (in fasted-state media) or micelles with diameters near 6.5 nm (in fed-state media) (5,63–66).

The extent of solubilization in mixed lipidic aggregates depends on colloidal properties such as lipid concentration, BS and PL composition, ratio of BS to PL, and structure. Further, drug properties such as size, charge, polarity, flexibility, and lipophilicity impact the partitioning into these colloids (5). Based on solubility profiling of more than 100 drug substances, Fagerberg and Bergström showed that molecules with a pH-dependent partition coefficient between octanol and water (log *D*) greater than 3 at pH 6.5 showed significantly higher solubility in FaSSIF than in blank buffer (i.e., same buffer species/concentration and pH but excluding BS and PL) (5). This result is intuitive; because the volume of FaSSIF lipidic aggregates/vesicles is about 0.1% of aqueous volume, 1000-fold partitioning into these structures enhance solubility by at least 2-fold. Based on these data, the solubility of lipophilic compounds (log *D* > 3) should always be assessed in a FaSSIF (any version) rather than a simple buffer to estimate solubilization that occurs *in vivo* (14,55,67–72).

For cases where the dose of drug exceeds *C*_s_ in blank buffer, then the increased solubilization in mixed lipidic aggregates would increase the extent of dissolution due to the increased solubilization capacity of the fluid. However, when the dose is less than *C*_s_ in blank buffer, the extent of dissolution with and without mixed lipidic aggregates would be expected to be similar. The rate of dissolution in the presence of mixed lipidic aggregates is influenced by a competing effect of *D*_eff_ and *C*_s_. While solubilization of drug in lipidic aggregates increases *C*_s_, it decreases *D*_eff_ due to an increase in the effective size of drug associated with mixed lipidic aggregates over that of unbound drug (73–76).

**Fig. 5:**
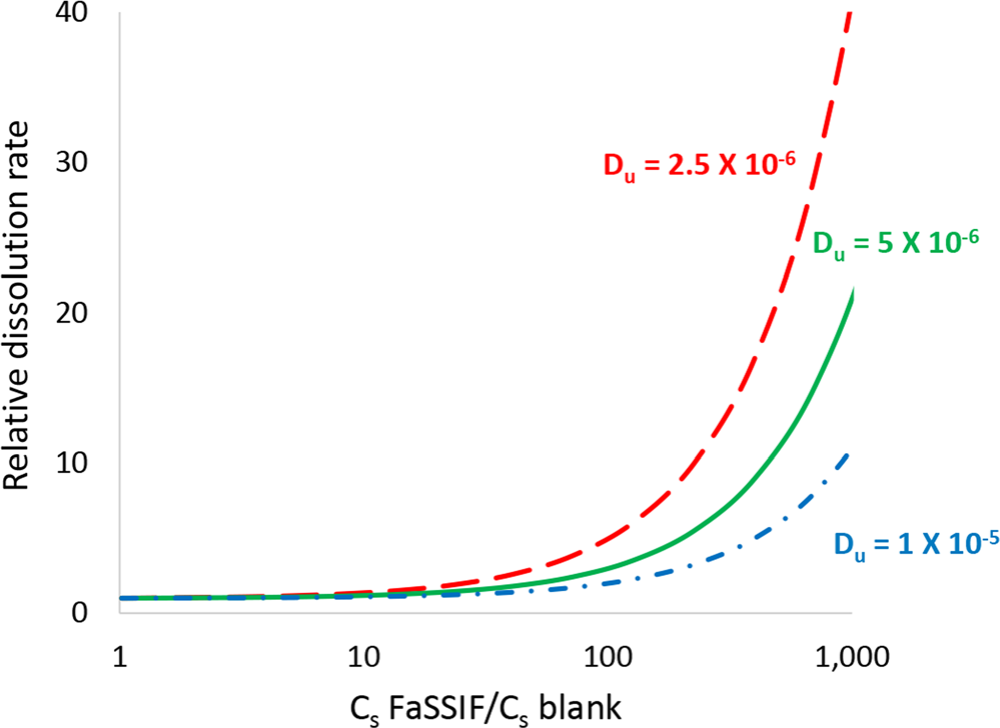
Calculated relative dissolution rate in FaSSIF *versus* blank buffer as a function of solubility ratio (e.g., solubility in FaSSIF/solubility in blank buffer) for drugs with different aqueous diffusion coefficients. Blank buffer comprises the same buffer species and concentration as FaSSIF, but excludes BS and PS. Assumes micelle diffusion coefficient = 1 × 10^−7^ cm^2^/s

Figure 5 shows the predicted relative dissolution rate in FaSSIF *versus* blank buffer as a function of the relative solubility in FaSSIF *versus* blank buffer. The basis for these calculations is described in Sect. 5 in the Electronic Supplementary Materials. As shown, a 10-fold increase in *C*_s_ compared with blank buffer is expected to lead to only a 1.2-fold increase in dissolution rate under sink conditions assuming *D*_eff_ = 5 × 10^−6^ cm^2^/s. Fagerberg and Bergström showed a fold increase in FaSSIF exceeding 10 only for compounds with log *D*_6.5_ values > 3. Therefore, only a minor difference in dissolution rate between FaSSIF and blank buffer would be expected for compounds with log *D* < 3 and *D*_eff_ = 5 × 10^−6^ cm^2^/s. More significant differences in dissolution rate between FaSSIF and blank buffer would be expected for compounds with much higher extents of solubilization in mixed lipidic aggregates. Assuming sink conditions and *D*_eff_ = 5 × 10^−6^ cm^2^/s, it would take a 50-fold increase in C_s_ compared to blank buffer to produce a 2-fold increase in dissolution rate, and a 450-fold increase in *C*_s_ compared with blank buffer to produce a 10-fold increase in dissolution rate.

#### Recommendations

We provide recommendations in Fig. 1 regarding addition of BS and PL in the dissolution medium. We select a log *D* > 3 as a minimum value for when to add BS and PL to the medium. While log *D* is not the sole determinant of solubilization in mixed lipidic aggregates, it serves as a convenient estimate (5). The extent of solubilization in mixed lipidic aggregates is also influenced by physicochemical properties as previously described. While some compounds with log *D* < 3 may have a fold increase in C_s_ compared to blank buffer higher than what was observed by Fagerberg and Bergström (i.e., > 10-fold), a fold increase below 50 still would not be expected to significantly impact dissolution rate assuming *D*_eff_ ≥ 5 × 10^−6^ cm^2^/s. BS and PL have been shown to improve wetting by lowering the interfacial tension between the dissolution medium and the drug. Therefore, inclusion of these components in the medium could be considered for compounds with log *D* < 3 that demonstrate poor wetting characteristics (77).

No single type and concentration of bile salts and phospholipids is expected to provide the best forecast of solubility in FaHIF for all drugs. Therefore, we recommend using the BS and PL types and concentrations of one of the standard fasted-state media, e.g., FaSSIF, FaSSIF-v2, or FaSSIF-v3 for intestinal media or FaSSGF for gastric media. While the original FaSSIF composition and FaSSIF-v2 contain only two bile components (lecithin and taurocholate), FaSSIF-v3 more closely resembles luminal composition with the incorporation of additional components, such as lecithin hydrolysis products and cholesterol (see Table III). For acids and bases, FaSSIF and FaSSIF-V2 both provide solubility values similar to those obtained in aspirated FaHIF (72). For neutral compounds, the FaSSIF seems to over predict the effect of solubilization, whereas the solubility values obtained in FaSSIF-V2 and the aspirated FaHIF are in better agreement (72). Fuchs and coworkers compared the solubility values of ten different model compounds in FaSSIF, FaSSIF-v2, and FaSSIF-v3, as well as in FaHIF. They found the differences in solubility values across *in vitro* media vary as a function of the drug, with some having equal solubility values in all three media and others showing increased solubility in one or two media. FaSSIF-v3 provided an equal or better forecast of solubility in FaHIF for eight of the compounds compared with FaSSIF and FaSSIF-v2. Evaluating the dissolution rate using all three compositions of bile components—as reflected by FaSSIF, FaSSIF-v2, and FaSSIF-v3—would provide an early indication of differences within and among individuals in dissolution screens.

## CONFIRMATION OF RECOMMENDATIONS

To confirm the recommendations, we performed in-house tests on eight model compounds and evaluated the recommendations against multiple reports in the literature. The study was narrowed to the impacts of buffer concentration and inclusion of BS and PL on dissolution rate since these factors have been less studied than pH impacts (4,26). In all cases, the recommendations were in good agreement with the results.

### In-house Experimental Evaluation

*In vitro* measurements were performed in house to confirm the recommendations. We determined the dissolution rate for eight model compounds that reflected different categories (e.g., acid/base/nonionizable; highly lipophilic *versus* modestly lipophilic). Experiments were performed under sink conditions using a μDISS Profiler (Pion Inc., Billerica, Massachusetts) in 10 to 20 mL of dissolution medium at 37 °C. Methods and a detailed explanation of the experimental confirmation are provided in Sect. 6 in the Electronic Supplementary Materials.

The in-house experimental evaluation showed that the recommendations correctly identified when a biorelevant buffer capacity or the addition of the bile salts/phospholipids to the medium would appreciably change the dissolution profiles of the compounds. For compounds for which biorelevant buffer capacity was expected to show no change in dissolution rate (i.e., < 2-fold difference), the difference in initial dissolution rate was 0.9–1.8-fold, whereas when a change was expected, the difference was 2.7- to 8.1-fold. For compounds for which the addition of BS and PL was expected to show no change in dissolution rate, the difference in initial dissolution rate was 1.0- to 1.1-fold, whereas when a change was expected, the difference in initial dissolution rate was 1.3- to 1.7-fold.

### Literature Evaluation

Literature evaluations assessing the recommendations herein were focused on studies that probed the effect of (1) buffer concentration on dissolution rate and (2) BS and PL on dissolution rate. As described below, both evaluations revealed good agreement with recommendations.

#### Effect of Buffer Capacity on Dissolution Rate

We evaluated multiple literature studies to determine the effect of buffer capacity on dissolution rate. In the first study, the dataset of acids published by Krieg and coworkers confirms the recommended guidelines for selection of biorelevant buffer capacity (59). They studied the effect of phosphate buffer concentration on dissolution rate for five different acids and one base in the rotating-disk dissolution apparatus at a pH of 6.5. All six compounds had *α* (acids) or *β* (bases) values less than 10 and showed an increase in flux at higher buffer concentrations within the range of ~ 3 to 50 mM. “Equivalent” phosphate buffer concentrations for these compounds ranged from less than 1 mM to 30 mM.

In the second study, Cristofoletti and Dressman used the approach of calculating an equivalent phosphate (5.0 mM) or equivalent maleate (2.2 mM) buffer to match dissolution rate of ibuprofen tablets in a physiological bicarbonate buffer at a pH of 6.7 (78–80). They found that a 5.0-mM phosphate concentration resulted in slower dissolution compared to a 13.5-mM phosphate concentration (i.e. FaSSIF-v3 buffer concentration), as well as better predicted *in vivo* performance differences of two different tablet formulations of ibuprofen.

In the third study, Hamed and coworkers demonstrated an increase in the dissolution rate of a weak acid, valsartan, as a function of buffer concentration in phosphate buffer (81). Within the first 5 min of the experiment, 36.3, 55.2, 72.3, and 82.9% of valsartan was released in 12.5-, 25-, 50-, and 100-mM buffer concentrations, respectively, despite maintaining a bulk pH of 6.8 throughout the duration of the experiment. Valsartan has a p*K*_a_ of 4.37 and an *α* value of 5.0 (82). These values meet the criteria for an expected effect of buffer capacity/concentration (p*K*_a_ < 6 and *α* < 10). The same authors showed an increase in the dissolution rate of a weak base, carvedilol, as a function of buffer concentration in phosphate buffer (83). The authors showed that approximately 34% of the dose was released after 60 min for a 6.25-mM buffer, whereas approximately 52 to 58% of the dose was released after 60 min for buffer concentrations ranging from 12.5 to 100 mM. The bulk pH of 6.8 was maintained within ± 0.05 units through 60 min. Carvedilol has a p*K*_a_ of 7.8 and *β* value of 5.7, which again meets the methodology criteria for bases showing buffer capacity-dependent dissolution (p*K*_a_ > 7 and *β* < 11) (83).

#### Effect of Bile Salts/Phospholipids on Dissolution Rate

For this evaluation, we looked at a literature report by Okazaki and coworkers, who performed dissolution experiments using suspensions of two neutral drugs—griseofulvin (Admet Predictor log *P* = 2.5) and Danazol (log *P* = 4.5)—in a USP 2 apparatus (76). They compared dissolution rates for each drug in buffers containing bile salts and lipids in the medium (sodium taurocholate concentrations of 3 (FaSSIF), 15 (FeSSIF), and 30 mM with sodium taurocholate:lecithin 4:1) to blank buffer. They found the increase in solubilization due to added bile salts and lipids in the medium was higher than the increase in initial dissolution rate in five out of six cases due to the decrease in effective diffusion coefficient. Griseofulvin showed a modest 1.5-fold increase in dissolution rate in FaSSIF compared to blank buffer, whereas the more lipophilic Danazol showed a 2.5-fold increase in dissolution rate. Additional researchers have reported similar results for other types for media, for example, using FaSSIF-dog and sodium dodecyl sulfate (75,84,85).

## *IN VITRO* TEST METHOD AND *IN SILICO* MODELING CONSIDERATIONS

While this tutorial focuses on selection of the optimal BDM, it is important to consider the impact of *in vitro* test methods and usefulness of *in silico* modeling. *In vitro* dissolution testing can serve a multitude of purposes through drug development and commercialization (23). The recommendations outlined in this tutorial are well suited to biorelevant dissolution testing. However, in some cases, they can also be useful for development of a QC or clinically relevant methods. QC methods typically require standard apparatuses with one compartment, commonly used buffers that do not contain BS or PL (but may contain synthetic surfactants) and standard medium volumes. In addition, QC methods for IR products tend to specify sink conditions with 80% release within a relatively short time frame, such as within 30 min. These methods must be low in cost, complexity, time, and labor. Since our recommendations suggest the simplest, yet accurate dissolution medium/media based upon drug properties, they work toward selection of a “QC-relevant” medium whenever possible; that is, when drug properties suggest that added medium cost and complexity are not critical for achieving biorelevant dissolution results. In this way, these guidelines provide a preliminary analysis in terms of understanding when a medium may meet the requirements of both a QC medium and a biorelevant medium. In addition, since our recommendations suggest inclusion of key properties and components expected to impact *in vivo* performance, they may also be useful for the development of dissolution media for clinically relevant dissolution methods.

The choice of dissolution apparatus such as single compartment, multicompartmental, or a system comprising an absorption compartment can significantly impact results (86). In addition, associated parameters such as fluid volume(s), stirring and related shear and hydrodynamics and transfer rates (if applicable) are important considerations (10,23,25).

*In silico* modeling can complement *in vitro* dissolution testing in facilitating understanding of the range in important physiological, drug substance and formulation properties impacting *in vivo* performance (87). For example, commercial software packages or in-house models can be used to study the impact of factors such as pH, buffer concentration, and mixed lipidic aggregate concentration on *in vitro* dissolution performance. Modeling can complement the approach of minimizing the number and complexity of media selected for *in vitro* dissolution testing by predicting these impacts *in silico*. Further, these *in vitro* predictions can be coupled with physiologically based modeling to further predict *in vivo* performance of the drug product of interest.

In addition, modeling could be used to further refine the recommendations in this tutorial. For example, one could integrate drug p*K*_a_, intrinsic solubility and log *D*/extent of solubilization into a dissolution model. This model could be solved for various types of dissolution media, providing relative dissolution rates as a function of several physiological and drug substance factors simultaneously.

## CONCLUSION

This tutorial presents a methodology to select a practical yet physiologically relevant dissolution medium for assessing dissolution of standard IR dosage forms administered to fasted humans. Recommendations are primarily suited toward biorelevant dissolution testing, which typically commences during early development and may continue through clinical testing and beyond. However, in some cases, they can also be useful for development of QC or clinically relevant methods.

This methodology is based upon the mechanisms by which drug substance physicochemical properties and human physiological characteristics influence *in vivo* dissolution of drug substances. The drug physicochemical properties needed to employ these recommendations can be predicted *a priori* using in-house models or commercial tools or be measured *in vitro* if further refinement is needed. While using a dissolution medium that mimics *in vivo* human intestinal fluid as closely as possible would be expected to provide the most biorelevant dissolution results, simplifying the medium has several advantages, such as reducing time and cost and increasing medium robustness. Finally, for biorelevant dissolution testing to be fully realized, these recommendations must be coupled with a physiologically relevant dissolution apparatus and associated testing parameters.

## ELECTRONIC SUPPLEMENTARY MATERIALS

ESM 1 (DOCX 431 kb)

## ABBREVIATIONS

*α*: p*K*_a_-log *S*_o_
*A*_s_: Surface area of the dissolving drug particles
BCS: Biopharmaceutics Classification System
BDM: Biorelevant dissolution medium
*β*: p*K*_w_-p*K*_a_-log *S*_o_
BS: Bile salts
*C*_b_: Measured bulk concentration
*C*_s_: Saturation solubility (concentration of non-ionized + ionized drug at saturation)
*C*_s_/*S*_o_: Relative solubility
*D*_eff_: Effective drug diffusivity in the dissolution medium
FaHIF: Fasted-state human intestinal fluid
FeHIF: Fed-state human intestinal fluid
FaSSIF: Fasted-state simulated intestinal fluid
FeSSIF: Fed-state simulated intestinal fluid
GI: Gastrointestinal
Log *D*: Logarithm of pH-adjusted partition coefficient between octanol and water
Log *D*_6.5_: Logarithm of pH-adjusted partition coefficient between octanol and water at pH = 6.5
Log *P*: Logarithm of the partition coefficient between octanol and water for a completely non-ionized molecule
pH: Negative logarithm of the hydrogen ion concentration
p*K*_a_: Negative logarithm of the acid dissociation constant
p*K*_w_: Negative logarithm of the water dissociation constant
PL: Phospholipids
*R*: Effective particle radius
SGF: Simulated gastric fluid
Sh: Sherwood number
SIF: Simulated intestinal fluid
*S*_o_: Intrinsic solubility
*t*: Time
USP: US Pharmacopeia
*V*_b_: Volume of bulk dissolution medium

## Footnotes

1 p*K*_w_ value of 13.6 at 37°C and saturated vapor pressure used in this tutorial (60).

## References

[1] USP (1955). The Pharmacopeia of the United States of America XV.

[2] USP (1960). The Pharmacopeia of the United States of America XVI.

[3] U.S. Department of Health and Human Services Food and Drug Administration Center for Drug Evaluation and Research (CDER). Waiver of In Vivo Bioavailability and Bioequivalence Studies for Immediate-Release Solid Oral Dosage Forms Based on a Biopharmaceutics Classification System Guidance for Industry. Silver Spring, MD; 2017.

[4] Amidon GL, Lennernas H, Shah VP, Crison JR (1995). A theoretical basis for a biopharmaceutic drug classification—the correlation of in-vitro drug product dissolution and in-vivo bioavailability. Pharm Res.

[5] Fagerberg JH, Bergstrom CAS (2015). Intestinal solubility and absorption of poorly water soluble compounds: predictions, challenges and solutions. Ther Deliv.

[6] Fuchs A, Dressman JB (2014). Composition and physicochemical properties of fasted-state human duodenal and Jejunal fluid: a critical evaluation of the available data. J Pharm Sci.

[7] Hens B, Tsume Y, Bermejo M, Paixao P, Koenigsknecht MJ, Baker JR, Hasler WL, Lionberger R, Fan J, Dickens J, Shedden K, Wen B, Wysocki J, Loebenberg R, Lee A, Frances A, Amidon G, Yu A, Benninghoff G, Salehi N, Talattof A, Sun D, Amidon GL (2017). Low buffer capacity and alternating motility along the human gastrointestinal tract: implications for in vivo dissolution and absorption of ionizable drugs. Mol Pharm.

[8] Koenigsknecht MJ, Baker JR, Wen B, Frances A, Zhang H, Yu A, Zhao T, Tsume Y, Pai MP, Bleske BE, Zhang X, Lionberger R, Lee A, Amidon GL, Hasler WL, Sun D (2017). In vivo dissolution and systemic absorption of immediate release ibuprofen in human gastrointestinal tract under fed and fasted conditions. Mol Pharm.

[9] Lindahl A, Ungell AL, Knutson L, Lennernäs H (1997). Characterization of fluids from the stomach and proximal jejunum in men and women. Pharm Res.

[10] Mudie DM, Amidon GL, Amidon GE (2010). Physiological parameters for oral delivery and in vitro testing. Mol Pharm.

[11] Pedersen PB, Vilmann P, Bar-Shalom D, Müllertz A, Baldursdottir S (2013). Characterization of fasted human gastric fluid for relevant rheological parameters and gastric lipase activities. Eur J Pharm Biopharm.

[12] Riethorst D, Baatsen P, Remijn C, Mitra A, Tack J, Brouwers J, Augustijns P (2016). An in-depth view into human intestinal fluid colloids: intersubject variability in relation to composition. Mol Pharm.

[13] Fuchs A, Leigh M, Kloefer B, Dressman JB (2015). Advances in the design of fasted state simulating intestinal fluids: FaSSIF-V3. Eur J Pharm Biopharm.

[14] Galia E, Nicolaides E, Horter D, Lobenberg R, Reppas C, Dressman JB (1998). Evaluation of various dissolution media for predicting in vivo performance of class I and II drugs. Pharm Res.

[15] Gray VA, Dressman JB (1996). Change of pH requirements for simulated intestinal fluid TS. Pharmacopeial Forum.

[16] Jantratid E, Janssen N, Reppas C, Dressman JB (2008). Dissolution media simulating conditions in the proximal human gastrointestinal tract: an update. Pharm Res.

[17] Khoshakhlagh P, Johnson R, Langguth P, Nawroth T, Schmueser L, Hellmann N, Decker H, Szekely NK (2015). Fasted-state simulated intestinal fluid “FaSSIF-C”, a cholesterol containing intestinal model medium for in vitro drug delivery development. J Pharm Sci.

[18] Psachoulias D, Vertzoni M, Goumas K, Kalioras V, Beato S, Butler J, Reppas C (2011). Precipitation in and supersaturation of contents of the upper small intestine after administration of two weak bases to fasted adults. Pharm Res.

[19] Vertzoni M, Dressman J, Butler J, Hempenstall J, Reppas C (2005). Simulation of fasting gastric conditions and its importance for the in vivo dissolution of lipophilic compounds. Eur J Pharm Biopharm.

[20] Riethorst D, Mols R, Duchateau G, Tack J, Brouwers J, Augustijns P (2016). Characterization of human duodenal fluids in fasted and fed state conditions. J Pharm Sci.

[21] Vertzoni M, Diakidou A, Chatzilias M, Söderlind E, Abrahamsson B, Dressman JB, Reppas C (2010). Biorelevant media to simulate fluids in the ascending colon of humans and their usefulness in predicting intracolonic drug solubility. Pharm Res.

[22] Augustijns P, Wuyts B, Hens B, Annaert P, Butler J, Brouwers J (2014). A review of drug solubility in human intestinal fluids: implications for the prediction of oral absorption. Eur J Pharm Sci.

[23] Grady H, Elder D, Webster GK, Mao Y, Lin Y, Flanagan T, Mann J, Blanchard A, Cohen MJ, Lin J, Kesisoglou F, Hermans A, Abend A, Zhang L, Curran D (2018). Industry’s view on using quality control, biorelevant, and clinically relevant dissolution tests for pharmaceutical development, registration, and commercialization. J Pharm Sci.

[24] Andreas CJ, Rosenberger J, Butler J, Augustijns P, McAllister M, Abrahamsson B, Dressman J (2018). Introduction to the OrBiTo decision tree to select the most appropriate in vitro methodology for release testing of solid oral dosage forms during development. Eur J Pharm Biopharm.

[25] Butler J, Hens B, Vertzoni M, Brouwers J, Berben P, Dressman J, Andreas CJ, Schaefer KJ, Mann J, McAllister M, Jamei M, Kostewicz E, Kesisoglou F, Langguth P, Minekus M, Müllertz A, Schilderink R, Koziolek M, Jedamzik P, Weitschies W, Reppas C, Augustijns P (2019). In vitro models for the prediction of in vivo performance of oral dosage forms: recent progress from partnership through the IMI OrBiTo collaboration. Eur J Pharm Biopharm.

[26] Butler JM, Dressman JB (2010). The developability classification system: application of biopharmaceutics concepts to formulation development. J Pharm Sci.

[27] Markopoulos C, Andreas CJ, Vertzoni M, Dressman J, Reppas C (2015). In-vitro simulation of luminal conditions for evaluation of performance of oral drug products: choosing the appropriate test media. Eur J Pharm Biopharm.

[28] Rosenberger J, Butler J, Muenster U, Dressman J (2019). Application of a refined developability classification system. J Pharm Sci.

[29] Taniguchi C, Kawabata Y, Wada K, Yamada S, Onoue S (2014). Microenvironmental pH-modification to improve dissolution behavior and oral absorption for drugs with pH-dependent solubility. Expert Opin Drug Deliv.

[30] Al-Gousous J, Ruan H, Blechar JA, Sun KX, Salehi N, Langguth P (2019). Mechanistic analysis and experimental verification of bicarbonate-controlled enteric coat dissolution: potential in vivo implications. Eur J Pharm Biopharm.

[31] Stewart A, Yates I, Mudie D, Pivette P, Goodwin A, Sarmiento A (2019). Mechanistic study of belinostat oral absorption from spray-dried dispersions. J Pharm Sci.

[32] Wang Y, Abrahamsson B, Lindfors L, Brasseur JG (2012). Comparison and analysis of theoretical models for diffusion-controlled dissolution. Mol Pharm.

[33] Wang Y, Abrahamsson B, Lindfors L, Brasseur JG (2015). Analysis of diffusion-controlled dissolution from polydisperse collections of drug particles with an assessed mathematical model. J Pharm Sci.

[34] Wang Y (2019). Brasseur JG.

[35] Avdeef A (2001). Physicochemical profiling (solubility, permeability and charge state). Curr Top Med Chem.

[36] Manallack DT (2009). The acid-base profile of a contemporary set of drugs: implications for drug discovery. SAR QSAR Environ Res.

[37] Tsume Y, Mudie DM, Langguth P, Amidon GE, Amidon GL (2014). The biopharmaceutics classification system: subclasses for in vivo predictive dissolution (IPD) methodology and IVIVC. Eur J Pharm Sci.

[38] Bergstrom CAS, Holm R, Jorgensen SA, Andersson SBE, Artursson P, Beato S (2014). Early pharmaceutical profiling to predict oral drug absorption: current status and unmet needs. Eur J Pharm Sci.

[39] Litou C, Vertzoni M, Goumas C, Vasdekis V, Xu W, Kesisoglou F (2016). Characteristics of the human upper gastrointestinal contents in the fasted state under hypo- and A-chlorhydric gastric conditions under conditions of typical drug–drug interaction studies. Pharm Res.

[40] Gao Y, Carr RA, Spence JK, Wang WW, Turner TM, Lipari JM, Miller JM (2010). A pH-dilution method for estimation of biorelevant drug solubility along the gastrointestinal tract: application to physiologically based pharmacokinetic modeling. Mol Pharm.

[41] Mann J, Dressman J, Rosenblatt K, Ashworth L, Muenster U, Frank K, Hutchins P, Williams J, Klumpp L, Wielockx K, Berben P, Augustijns P, Holm R, Hofmann M, Patel S, Beato S, Ojala K, Tomaszewska I, Bruel JL, Butler J (2017). Validation of dissolution testing with biorelevant media: an OrBiTo study. Mol Pharm.

[42] Berben P, Ashworth L, Beato S, Bevernage J, Bruel JL, Butler J, Dressman J, Schäfer K, Hutchins P, Klumpp L, Mann J, Nicolai J, Ojala K, Patel S, Powell S, Rosenblatt K, Tomaszewska I, Williams J, Augustijns P (2019). Biorelevant dissolution testing of a weak base: Interlaboratory reproducibility and investigation of parameters controlling in vitro precipitation. Eur J Pharm Biopharm.

[43] Berlin M, Ruff A, Kesisoglou F, Xu W, Wang MH, Dressman JB (2015). Advances and challenges in PBPK modeling—analysis of factors contributing to the oral absorption of atazanavir, a poorly soluble weak base. Eur J Pharm Biopharm.

[44] Bhattachar SN, Perkins EJ, Tan JS, Burns LJ (2011). Effect of gastric pH on the pharmacokinetics of a BCS class II compound in dogs: utilization of an artificial stomach and duodenum dissolution model and GastroPlus, (TM) simulations to predict absorption. J Pharm Sci.

[45] Carino SR, Sperry DC, Hawley M (2006). Relative bioavailability estimation of carbamazepine crystal forms using an artificial stomach-duodenum model. J Pharm Sci.

[46] Carino SR, Sperry DC, Hawley M (2010). Relative bioavailability of three different solid forms of PNU-141659 as determined with the artificial stomach-duodenum model. J Pharm Sci.

[47] Ding X, Gueorguieva I, Wesley JA, Burns LJ, Coutant CA (2015). Assessment of in vivo clinical product performance of a weak basic drug by integration of in vitro dissolution tests and physiologically based absorption modeling. AAPS J.

[48] Gu CH, Rao D, Gandhi RB, Hilden J, Raghavan K (2005). Using a novel multicompartment dissolution system to predict the effect of gastric pH on the oral absorption of weak bases with poor intrinsic solubility. J Pharm Sci.

[49] Kostewicz ES, Wunderlich M, Brauns U, Becker R, Bock T, Dressman JB (2004). Predicting the precipitation of poorly soluble weak bases upon entry in the small intestine. J Pharm Pharmacol.

[50] Matsui K, Tsume Y, Amidon GE, Amidon GL (2015). In vitro dissolution of fluconazole and dipyridamole in gastrointestinal simulator (GIS), predicting in vivo dissolution and drug-drug interaction caused by acid-reducing agents. Mol Pharm.

[51] Sperry DC, Hawley M (2000). Dynamic artificial stomach and intestine model to evaluate the bioavailability of drugs and formulations. Abstr Pap Am Chem Soc.

[52] Takeuchi S, Tsume Y, Amidon GE, Amidon GL (2014). Evaluation of a three compartment in vitro gastrointestinal simulator dissolution apparatus to predict in vivo dissolution. J Pharm Sci.

[53] Tsume Y, Takeuchi S, Matsui K, Amidon GE, Amidon GL (2015). In vitro dissolution methodology, mini-gastrointestinal simulator (mGIS), predicts better in vivo dissolution of a weak base drug, dasatinib. Eur J Pharm Sci.

[54] de la Cruz Moreno MP, Oth M, Deferme S, Lammert F, Tack J, Dressman J (2006). Characterization of fasted-state human intestinal fluids collected from duodenum and jejunum. J Pharm Pharmacol.

[55] Persson EM, Gustafsson AS, Carlsson AS, Nilsson RG, Knutson L, Forsell P, Hanisch G, Lennernäs H, Abrahamsson B (2005). The effects of food on the dissolution of poorly soluble drugs in human and in model small intestinal fluids. Pharm Res.

[56] Kalantzi L, Goumas K, Kalioras V, Abrahamsson B, Dressman JB, Reppas C (2006). Characterization of the human upper gastrointestinal contents under conditions simulating bioavailability/bioequivalence studies. Pharm Res.

[57] McGee LC, Hastings AB (1942). The carbon dioxide tension and acid-base balance of jejunal secretions in man. J Biol Chem.

[58] Amaral Silva D, Al-Gousous J, Davies NM, Bou Chacra N, Webster GK, Lipka E (2019). Simulated, biorelevant, clinically relevant or physiologically relevant dissolution media: the hidden role of bicarbonate buffer. Eur J Pharm Biopharm.

[59] Krieg BJ, Taghavi SM, Amidon GL, Amidon GE (2015). In vivo predictive dissolution: comparing the effect of bicarbonate and phosphate buffer on the dissolution of weak acids and weak bases. J Pharm Sci.

[60] CRC handbook of chemistry and physics: a ready-reference of chemical and physical data, 77th edn. Edited by David R. Lide (National Institute of Standards and Technology). CRC Press LLC: Journal of the American Chemical Society. Boca Raton, FL: Publisher Taylor & Francis Inc.; 1996.

[61] Pepin XJH, Sanderson NJ, Blanazs A, Grover S, Ingallinera TG, Mann JC (2019). Bridging in vitro dissolution and in vivo exposure for acalabrutinib. Part I. mechanistic modelling of drug product dissolution to derive a P-PSD for PBPK model input. Eur J Pharm Biopharm.

[62] Muellertz A, Fatouros DG, Smith JR, Vertzoni M, Reppas C (2012). Insights into intermediate phases of human intestinal fluids visualized by atomic force microscopy and cryo-transmission electron microscopy ex vivo. Mol Pharm.

[63] Boni JE, Brickl RS, Dressman J, Pfefferle ML (2009). Instant FaSSIF and FeSSIF-biorelevance meets practicality. Dissolut Technol.

[64] Kloefer B, van Hoogevest P, Moloney R, Kuentz M, Leigh MLS, Dressman J (2010). Study of a standardized taurocholate-lecithin powder for preparing the biorelevant media FeSSIF and FaSSIF. Dissolut Technol.

[65] Nawroth T, Buch P, Buch K, Langguth P, Schweins R (2011). Liposome formation from bile salt-lipid micelles in the digestion and drug delivery model FaSSIF mod estimated by combined time-resolved neutron and dynamic light scattering. Mol Pharm.

[66] Sugano K, Okazaki A, Sugimoto S, Tavornvipas S, Omura A, Mano T (2007). Solubility and dissolution profile assessment in drug discovery. Drug Metab Pharmacokinet.

[67] Clarysse S, Brouwers J, Tack J, Annaert P, Augustijns P (2011). Intestinal drug solubility estimation based on simulated intestinal fluids: comparison with solubility in human intestinal fluids. Eur J Pharm Sci.

[68] Fadda HM, Sousa T, Carlsson AS, Abrahamsson B, Williams JC, Kumar D, Basit AW (2010). Drug solubility in luminal fluids from different regions of the small and large intestine of humans. Mol Pharm.

[69] Fagerberg JH, Al-Tikriti Y, Ragnarsson G, Bergstrom CAS (2012). Ethanol effects on apparent solubility of poorly soluble drugs in simulated intestinal fluid. Mol Pharm.

[70] Fagerberg JH, Tsinman O, Sun N, Tsinman K, Avdeef A, Bergstrom CAS (2010). Dissolution rate and apparent solubility of poorly soluble drugs in biorelevant dissolution media. Mol Pharm.

[71] Ottaviani G, Gosling DJ, Patissier C, Rodde S, Zhou L, Faller B (2010). What is modulating solubility in simulated intestinal fluids?. Eur J Pharm Sci.

[72] Soderlind E, Karlsson E, Carlsson A, Kong R, Lenz A, Lindborg S (2010). Simulating fasted human intestinal fluids: understanding the roles of lecithin and bile acids. Mol Pharm.

[73] Amidon GE, Higuchi WI, Ho NFH (1982). Theoretical and experimental studies of transport of micelle-solubilized solutes. J Pharm Sci.

[74] Balakrishnan A, Rege BD, Amidon GL, Polli JE (2004). Surfactant-mediated dissolution: contributions of solubility enhancement and relatively low micelle diffusivity. J Pharm Sci.

[75] Jinno J, Oh DM, Crison JR, Amidon GL (2000). Dissolution of ionizable water-insoluble drugs: the combined effect of pH and surfactant. J Pharm Sci.

[76] Okazaki A, Mano T, Sugano K (2008). Theoretical dissolution model of poly-disperse drug particles in biorelevant media. J Pharm Sci.

[77] Bakatselou V, Oppenheim RC, Dressman JB (1991). Solubilization and wetting effects of bile salts on the dissolution of steroids. Pharm Res.

[78] Cristofoletti R, Dressman JB (2016). Matching phosphate and maleate buffer systems for dissolution of weak acids: equivalence in terms of buffer capacity of bulk solution or surface pH?. Eur J Pharm Biopharm.

[79] Cristofoletti R, Dressman JB (2016). FaSSIF-V3, but not compendial media, appropriately detects differences in the peak and extent of exposure between reference and test formulations of ibuprofen. Eur J Pharm Biopharm.

[80] Cristofoletti R, Dressman JB (2017). Dissolution methods to increasing discriminatory power of in vitro dissolution testing for ibuprofen free acid and its salts. J Pharm Sci.

[81] Hamed R (2018). Physiological parameters of the gastrointestinal fluid impact the dissolution behavior of the BCS class IIa drug valsartan. Pharm Dev Technol.

[82] Hamed R, Alnadi SH (2018). Transfer behavior of the weakly acidic BCS class II drug valsartan from the stomach to the small intestine during fasted and fed states. AAPS PharmSciTech.

[83] Hamed R, Awadallah A, Sunoqrot S, Tarawneh O, Nazzal S, AlBaraghthi T (2016). pH-dependent solubility and dissolution behavior of carvedilol—case example of a weakly basic BCS class II drug. AAPS PharmSciTech.

[84] Sheng JJ, Kasim NA, Chandrasekharan R, Amidon GL (2006). Solubilization and dissolution of insoluble weak acid, ketoprofen: effects of pH combined with surfactant. Eur J Pharm Sci.

[85] Takano R, Furumoto K, Shiraki K, Takata N, Hayashi Y, Aso Y, Yamashita S (2008). Rate-limiting steps of oral absorption for poorly water-soluble drugs in dogs; prediction from a miniscale dissolution test and a physiologically-based computer simulation. Pharm Res.

[86] Kostewicz ES, Abrahamsson B, Brewster M, Brouwers J, Butler J, Carlert S, Dickinson PA, Dressman J, Holm R, Klein S, Mann J, McAllister M, Minekus M, Muenster U, Müllertz A, Verwei M, Vertzoni M, Weitschies W, Augustijns P (2014). In vitro models for the prediction of in vivo performance of oral dosage forms. Eur J Pharm Sci.

[87] Kostewicz ES, Aarons L, Bergstrand M, Bolger MB, Galetin A, Hatley O, Jamei M, Lloyd R, Pepin X, Rostami-Hodjegan A, Sjögren E, Tannergren C, Turner DB, Wagner C, Weitschies W, Dressman J (2014). PBPK models for the prediction of in vivo performance of oral dosage forms. Eur J Pharm Sci.

