# Supplementary material for: Selection of In Vivo Predictive Dissolution Media Using Drug Substance and Physiological Properties
Source: AAPS J. 2020 Jan 27;22(2):34. doi: 10.1208/s12248-020-0417-8 (PMC6985051; doi:10.1208/s12248-020-0417-8)
Supplement: Supplementary file 1 — (DOCX 431 kb) [file 12248_2020_417_MOESM1_ESM.docx]

Selection of *in vivo* predictive dissolution media using drug substance and physiological properties

**Supplementary Materials**

## Section 1. Calculating Relative Solubility

A useful parameter for comparing solubility values at pH values representative of different regions of the gastrointestinal (GI) tract is the “relative solubility”, which is equal to the total saturation solubility (C_s_) divided by the intrinsic solubility (S_o_). Here, C_s_ equals the solubility of ionized and nonionized drug forms and S_o_ equals the solubility of the nonionized drug form. The relative solubility can be calculated as a function of the fluid pH and the negative log of the acid dissociation constant (pK_a_) for the drug for monoprotic compounds (1). See **Equation S1a** for a monoprotic weak acid and **Equation S1b** for a monoprotic weak base.

$\frac{C_{s}}{S_{0}}=\left( 1+{10}^{\left( pH-pK_{a} \right)} \right)$ **(S1a)**

and

$\frac{C_{s}}{S_{0}}=\left( 1+{10}^{\left( pK_{a}-pH \right)} \right).$ **(S1b)**

**Figures S1** shows the calculated relative solubilities for monoprotic weak acids and bases as a function of pK_a_ and pH according to **Equation S1a** and **S1b**, respectively.

**Figure S1.** Calculated relative solubility (C_s_/S_o_) for monoprotic weak acids (top) and weak bases (bottom) as a function of pK_a_ and pH according to **Equation S1a** and **S1b**, respectively. These calculations assume a constant pH that is not impacted by dissolved drug.

**Figure S2.** Calculated C_s_ at pH 7 relative to C_s_ at pH 5.5 (monoprotic weak acids) and calculated C_s_ at pH 5.5 relative to C_s_ at pH 7 (monoprotic weak bases) as a function of pK_a_. These calculations assume a constant pH that is not impacted by dissolved drug.

## Section 2. Relative Dissolution Rate versus Buffer Concentration/Capacity

### Calculated relative dissolution rates at pH 5.5 and 7

**Figure S3** (acids) and **Figure S4** (bases) show calculated surface-area-normalized relative dissolution rates in a pH 5.5 and 7 phosphate buffer as a function of α or β. The relative dissolution rate is the calculated dissolution rate at high buffer concentration (50 mM) divided by the calculated dissolution rate at low buffer concentration (1 mM). When the relative dissolution rate equals unity, no differences in rate are expected over this range in buffer concentration.

**Figure S3**. Relative calculated surface-area normalized dissolution rate for a monoprotic weak acid at pH 5.5 (top) and pH 7 (bottom) as a function of α. Dashed line represents a factor of 2 difference.

**Figure S4**. Relative calculated surface-area normalized dissolution rate for a monoprotic weak base at pH 5.5 (top) and pH 7 (bottom) as a function of β. Dashed line represents a factor of 2 difference.

### Calculated relative dissolution rates versus infinite buffer capacity

In **Figure S5**, dissolution rate is normalized to the maximum dissolution rate (i.e., when no pH change occurs at the solid-particle surface as a drug dissolves) versus the fluid buffer capacity relative to the concentration of ionized species at the starting pH. The theoretical concentration of ionized species at the starting pH is dependent upon the intrinsic solubility and pK_a_ of the drug and the starting pH of the buffer, whereas the buffer capacity is calculated using the Van Slyke equation (i.e., as a function of buffer concentration, buffer pK_a_, and starting pH). As shown in **Figure S5**, the dissolution rate from the solid-particle surface will be lower than the dissolution rate occurring in a buffer with infinite buffer capacity when 1) the buffer capacity is within two orders of magnitude of the concentration of ionized drug species in solution at the starting pH, and 2) pH - pK_a_  > -1 (weak acids) or (pK_a_ – pH) > -1 (weak bases), where pH equals the starting pH of the buffer. When the first criterion is met, the pH at the solid-liquid interface (i.e., the “surface pH”) of the dissolving drug particle can range between the starting pH and fall as low as two pH units below (weak acid) or above (weak base) the pK_a_ of the compound. When the second criterion is met, the maximum percent ionized drug is 10% or greater. For poorly buffered systems, the percent ionization decreases as the pH drifts below (for weak acids) or above the pK_a_ (for weak bases), resulting in a decrease in solubility and dissolution rate.

**Figure S5** shows the predicted surface-area-normalized relative dissolution rate of a monoprotic drug (with a given intrinsic solubility and pK_a_) dissolving in a monoprotic buffer (at a given pH with a given pK_a_ and buffer concentration). The y axis represents the predicted dissolution rate in the given buffer divided by the predicted dissolution rate in a buffer at the same pH, but at infinite buffer capacity. The x axis represents the logarithm of buffer capacity divided by the concentration of ionized drug at the starting pH, where buffer capacity is calculated using the Van Slyke equation and the concentration of ionized drug at starting pH = S_0_(10^pH-pKa^) for acids and = S_0_(10^pKa-pH^) for bases. These calculations assume an aqueous diffusion coefficient of drug of 7.9 × 10^-6^ and a diffusion layer thickness = 30 µm. The shaded area represents the region in which buffer capacity is expected to have a significant impact on dissolution rate.


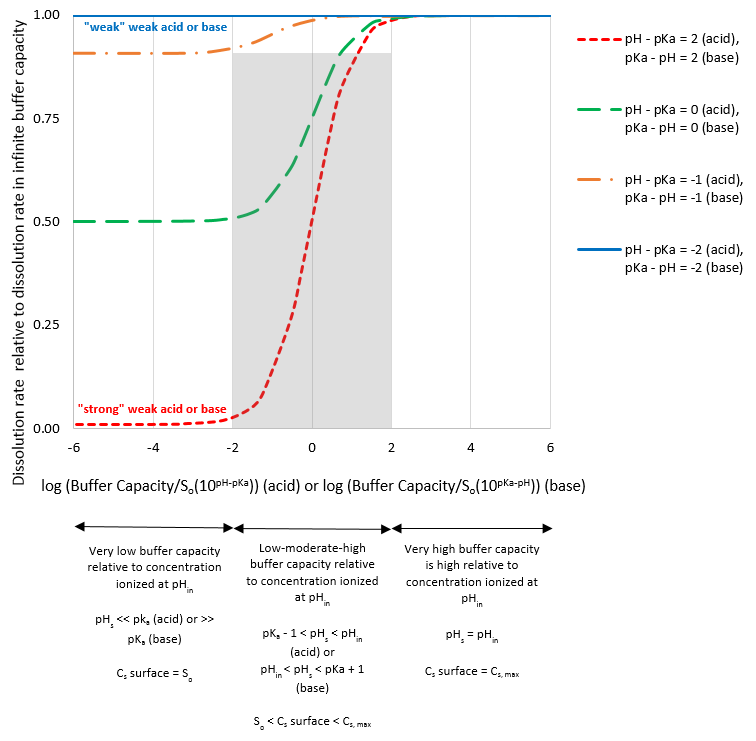


**Figure S5.** Predicted surface-area-normalized relative dissolution rate of a monoprotic drug dissolving in a monoprotic buffer**.** pH_s_ = pH at the surface of the solid-liquid interface of the dissolving drug substance, pH_in_ = starting pH in the bulk solution, S_0_ = intrinsic solubility of the drug substance, C_s_ = total saturation solubility at the solid-liquid interface of the dissolving drug substance, C_s,max_ = maximum total saturation solubility at the solid-liquid interface of the dissolving drug substance in the given buffer system (i.e., at starting pH).

## Section 3. Equivalent Maleate Buffer Concentrations at pH 5.5.

**Figure S6.** Equivalent pH 5.5 maleate buffer to match a 4 mM bicarbonate (16.7 mM total buffer concentration) buffer for a weak acid (top) or weak base (bottom). Assumes drug diffusivity = 7.9 X 10^-6^ cm^2^/s. Assumes diffusion layer thickness = 30 µm.

## Section 4. pH and Buffer Capacity Considerations for Polyprotic Drugs

The pH and buffer capacity recommendations provided in this tutorial are based upon monoprotic weak acids and bases (i.e. molecule contains a single pK_a_). A drug is considered a monoprotic weak acid when it has an acidic pK_a_ < 8. A drug is considered a monoprotic weak base when it has a pK_a_ > 1. Polyprotic weak acids have at least two acidic pK_a_s < 8, while polyprotic weak bases have at least two pK_a_s > 1. Zwitterions have at least one acidic pK_a_ < 8 and at least one basic pK_a_ >1. Recommendations for how to apply these recommendations to polyprotic weak acids and bases and zwitterions are described below.

### Selection of pH

pH recommendations in this tutorial are based upon whether a difference in solubility and therefore dissolution rate and/or precipitation is expected as a function of the pKa of a monoprotic weak acid or base and the physiological pH range in the fasted human. While not provided in this tutorial, the reader can use software such as ADMET Predictor^TM^ (Simulations Plus, Lancaster, CA) or chemicalize.com (ChemAxon Ltd), for example, to visualize the pH-ionization and pH-solubility profiles of a given polyprotic acid, polyprotic base or ampholyte. The pH-solubility profile can be used to determine over what pH range solubility (and therefore dissolution rate and/or precipitation) is expected to differ significantly. If the drug is highly non-ionized and solubility is at or near the intrinsic solubility in the pH range 1-3 of the stomach, then the drug can be categorized as a weak acid. If at least a 2-fold difference in solubility is expected over a pH range of 5.5 – 7, then the reader can follow the recommendations for when pK_a_ ≤ 7. If the drug is highly ionized and solubility is much higher than the intrinsic solubility (i.e. by 10-100-fold), then the drug can be categorized as a weak base. If at least a 2-fold difference in solubility is expected over a pH range of 5.5 – 7, then the reader can follow the recommendations for when pK_a_ ≥ 5.5.

In absence of determining the pH solubility profile, the following method can also be used for selecting pH for polyprotic acids or bases. For polyprotic bases, the recommendation for when pKa is ≥ 5.5 should be followed if at least one pK_a_ ≥ 5.5. For polyprotic acids the recommendation for when pKa ≤ 7 should be followed if at least one pK_a_ ≤ 7. However, this approach is not recommended for ampholytes, since the extent of ionization and therefore the solubility as a function of pH is less predictable.

### Selection of buffer capacity

For polyprotic acids, if at least one pK_a_ is less than the cutoff of 6, then dissolved drug may impact the solid surface pH when α is below the cutoff of 11. The value of α can be calculated using the lowest (i.e. strongest) pKa. For polyprotic weak bases, if at least one pK_a_ is greater than the cutoff of 7, then dissolved drug may impact the solid surface pH if β is below the cutoff of 12. The value of β can be calculated using the highest (i.e. strongest) pK_a_. This approach can also be applied to acidic and basic pK_a_s of ampholytes. If one or multiple pK_a_s suggest an impact of drug on solid surface pH then calculating an equivalent buffer is recommended.

## Section 5. Estimating the Relative Dissolution Rate in Fasted-State Simulated Intestinal Fluid (FaSSIF) Versus Blank Buffer

When drug is associated with mixed lipidic aggregates, the effective diffusion coefficient (D_eff_) accounts for that of unbound drug and drug in mixed lipidic aggregates/micelles. Assuming the main species in solution are unbound drug and drug in mixed lipidic aggregates, D_eff_ can be estimated using

$D_{eff}=D_{u}\cdot f_{u}+D_{m}\cdot f_{m}$ , **(S2)**

where D_u_ is the average diffusion coefficient of the unbound drug species, f_u_ is the fraction of the unbound drug species, D_m_ is the average diffusion coefficient of the micellar species, and f_m_ is the fraction of drug in micellar species (2-4).

Using the Stokes-Einstein equation to estimate the diffusion coefficient based on the average diameter of the vesicles present in FaSSIF (~45 nm), D_m_ = 1 x 10^-7^ cm^2^/s for these vesicles (this value has also been confirmed experimentally using dynamic light scattering) (5). The diffusion coefficient of the unbound drug can be estimated using empirical equations (e.g., the Hayduk-Laudie equation) and typically falls within the range of 1 x 10^-6^ to 1 x 10^-5^ (6). Assuming sink conditions and equivalent values of diffusion-layer thickness (δ), the dissolution rate of drug in a medium containing bile salts and phospholipids relative to that of a blank medium can be estimated as

$\boldsymbol{Relative dissolution rate=}\frac{\boldsymbol{C}_{\boldsymbol{s,FaSSIF}}}{\boldsymbol{C}_{\boldsymbol{s,blank}}}\boldsymbol{*}\frac{\boldsymbol{D}_{\boldsymbol{eff}}}{\boldsymbol{D}_{\boldsymbol{u}}}$ **(S3)**

Assumptions implicit in this equation are beyond the scope of this paper, but are discussed in other papers (7).

## Section 6. In-House Experimental Confirmation of Recommendations

To confirm the recommendations regarding buffer capacity and log D, we performed in-house tests on eight model compounds.

Materials and Methods

**Table S1** gives information on the eight model compounds tested. All compounds were purchased from Sigma-Aldrich (St. Louis, Missouri) or Spectrum Chemical (New Brunswick, New Jersey) and used as received. All dissolution measurements were performed using suspension methodologies in the µDISS Profiler™ (Pion Inc., Billerica, Massachusetts). The experiments were performed in 10 to 20 mL of dissolution medium at 37°C with stirring at 100 rpm. Dissolution testing was performed at pH 6.5 in four different buffers for each compound, except atenolol. These buffers included 1) 50 mM phosphate buffer, 2) 50 mM phosphate containing FaSSIF-v1 bile salts and lipids, 3) a low concentration ‘equivalent’ phosphate and 4) a low concentration ‘equivalent’ phosphate containing FaSSIF-v1 bile salts and lipids. The impact of log D was not determined for the very low log D (-2.8) compound, atenolol. This compound was only evaluated in buffers 1) and 3).

The two laboratories used slightly different approaches to produce the suspensions. Danazol, prednisolone, tamoxifen, and atenolol were studied at Uppsala University. Uppsala used a recent method based on controlled suspensions for danazol, prednisolone and tamoxifen (8). In this method, each drug was balled milled in a low-buffer-capacity medium consisting of 1% (w/w) polyvinyl pyrrolidone (PVP) K30 and 0.2% (w/w) sodium dodecyl sulfate (SDS). A disc method instead of the controlled suspensions method was used for atenolol due to its high solubility at a bulk pH of 6.5 (9). Discs (5 mg) were compressed at a pressure of 80 kg into Pion disc sets (3-mm cavity) with a surface area of 0.0707 cm^2^. The disc method allowed for a much smaller surface area and therefore decreased dissolution rate compared to the controlled suspensions method. This increased time allowed for enough data points to calculate the dissolution rate. Each dissolution assay was performed under sink conditions except for danazol. For danazol experiments, slightly non-sink conditions were used to allow accurate determination of the concentration, since ultraviolet (UV) absorbance values were too low for accurate measurements at concentrations representative of sink conditions.

Dipyridamole, ketoconazole, ibuprofen, and flufenamic acid were studied at Lonza using suspensions produced by adding the bulk powder to 0.5% (w/w) methylcellulose in water. The samples were vortexed to produce a homogenous suspension. The particle sizes in these suspensions were larger than those in Uppsala University tests, so the measurement time was extended to 60 minutes for most compounds.

Dissolution rate was determined by calculating the slope of the initial linear portion of the dissolution-time profile for each compound except Ibuprofen. The difference in dissolution rate between two different media was determined by taking the ratio of the two rates. For Ibuprofen, the time to dissolve 80% of the dose was determined for each medium, and the ratio of the two times was determined to compare media. This method was used since dissolution was rapid (100% dissolved in less than 1 minute) in the 50-mM buffer, which led to insufficient time points during the initial linear portion of the dissolution-time profile.

Results and Discussion

**Table S2** shows the predicted and experimental differences in dissolution rate as a function of buffer concentration and addition of bile salts and phospholipids for each model drug. The experimental dissolution curves are presented in **Figure S7 and S8**. The experimental evaluation showed that the recommendations correctly identified when a biorelevant buffer capacity and addition of the bile salts and phospholipids would appreciably change the dissolution profiles of the compounds, as discussed below.

***Acids***

Ibuprofen was selected as a non-lipophilic weak acid with low pK_a_, while flufenamic acid was chosen as a relatively lipophilic weak acid, also with a low pK_a_. For these compounds the dissolution rate increased from the calculated “equivalent” buffer concentration (3.5 mM and 0.4 mM, respectively) to a buffer concentration of 50 mM, as expected. For ibuprofen, a 7-fold increase in dissolution rate was expected at a concentration of 50 mM relative to 3.5 mM. Without bile salts/phospholipids (BS/PL) the experimental time to reach 80% dissolved was 7.4- fold higher in 50 mM compared to 3.5 mM buffer. This difference was 8.1-fold in the presence of BS/PL. For flufenamic acid, a 2 – 3-fold difference was expected between a 50 mM and 0.4 mM buffer. A 2.7-fold difference was observed with BS/PL and a 2.9-fold difference was observed without BS/PL. With a calculated log D_6.5_ of 1.7, ibuprofen showed a 1.1-fold difference in 50 mM buffer and no difference in 3.5 mM buffer. Flufenamic acid showed a 1.3-fold difference at a concentration of 50 mM, and a 1.1-fold difference at a concentration of 0.4 mM. With a calculated log D_6.5_ of 3.7, the measured solubility ratio in FaSSIF/blank buffer was 1.9, which resulted in a calculated dissolution rate ratio of 1.0 (10).

***Bases***

Dipyridamole and ketoconazole were chosen as a relatively lipophilic weak bases with relatively low pK_a_s. As expected, no difference in dissolution rate was observed for dipyridamole in equivalent (0.2-mM) buffer versus 50-mM buffer. A very slight increase in dissolution rate was observed with addition of bile salts and phospholipids (factor of 1.4 in both 0.2-mM and 50-mM media). Dipyridamole has a measured solubility ratio in FaSSIF/blank buffer in the range of 1.8 to 3.5, which results in a calculated dissolution rate ratio of 1.0 (10). Ketoconazole showed nearly identical dissolution rates in all four buffers with the exception of a slightly diminished rate in the blank equivalent (0.2-mM) buffer. When comparing calculated dissolution rates within the first 2 minutes of the experiments, the impact of buffer concentration was 0.9 with bile salts/phospholipids present and 1.8 without bile salts/phospholipids present (no difference was expected). The differences in calculated rates due to the presence of bile salts/phospholipids was 0.7 for 50-mM buffer and 1.5 for 0.2-mM buffer (a minor difference was expected). Results for the impact of bile salts/phospholipids are in line with a measured solubility ratio in the range of about 1.8 to 5 (10), leading to an expected 1.1-fold increase in dissolution rate in FaSSIF compared with blank buffer.

Atenolol was chosen as a non-lipophilic weak base with a high pK_a_. Since Atenolol is classified as BCS 1, it has a high S_o_. Therefore, while β < 11, surface pH is calculated to increase to a high and similar extent in both 50 mM and 1 mM buffer. Therefore, the difference in calculated dissolution rates is less than 2-fold. Atenolol was predicted to show a 1.7-fold higher flux at 50 mM than in the equivalent buffer. The observed difference was 1.3-fold.

Finally, tamoxifen was selected as a model compound reflecting a relatively lipophilic base with a high pK_a_. For this compound, no significant difference was expected between equivalent buffer and 50-mM buffer. Experimental results demonstrated a 1.1-fold difference in the presence of BS/PL and no difference in the absence of BS/PL. A 1.4-fold increase in dissolution rate was expected in FaSSIF versus blank buffer (experimental solubility ratio of 26) (10). Experimental findings agreed with expectations, showing a 1.4-fold difference in 50 mM buffer, and a 1.6-fold difference in 0.3 mM buffer.

***Neutral compounds***

Danazol (log P/log D_6.5_ > 3) and prednisolone (log P/ log D_6.5_ < 3) were selected as two model compounds to evaluate the effect of addition of bile salts and phospholipids. For these compounds, no difference in dissolution rate as a function of buffer concentration was expected. This expectation was confirmed as evidenced by a 0.9-fold and 1.1-fold difference in the presence and absence of BS/PL, respectively, for danazol, and a 0.9-fold difference both in the presence and absence of BS/PL for prednisolone. As a non-lipophilic compound, prednisolone was not expected to show increased dissolution rate when exposed to bile salts and phospholipids, and no experimental difference was observed. Danazol, which is highly lipophilic, was expected to show an increased dissolution rate in the presence of BS/PL. The experiments confirmed an increase in dissolution rate of 1.7-fold in both 50 mM and 10 mM buffer.

**Table S1.** Model compounds tested for confirmation of recommendations

| **Compound^a^** | **Compound Type** | **pK_a_** | **log D_6.5_** | **S_o_ (µg/mL)** |
| --- | --- | --- | --- | --- |
| Atenolol | Base | 9.5^b^ | -2.8^c^ | 13,500^b^ |
| Dipyridamole | Base | 6.2^d^ | 3.6^e^ | 14.5^d^ |
| Ketoconazole | Base | 6.1^f^ | 4.2^g^ | 3.7^f^ |
| Tamoxifen | Base | 8.5^h^ | 4.5^i^ | 0.04^j^ |
| Flufenamic acid | Acid | 4.6^k^ | 3.7^l^ | 1.3^m^ |
| Ibuprofen | Acid | 4.4^n^ | 1.7^o^ | 68^n^ |
| Danazol | Neutral | - | 4.5^p^ | 0.5^q^ |
| Prednisolone | Neutral | - | 1.6^r^ | 396^j^ |

| ^a^ Abbreviations used: partition coefficient between octanol and water at pH 6.5 (log D_6.5_); intrinsic solubility (S_O_).  ^b^ Reference (11)  ^c^ log P from reference (12). Calculated log D_6.5_ using log P and pK_a_.  ^d^ From reference (13).  ^e^ log P from Reference (14). Calculated log D_6.5_ using log P and pK_a_.  ^f^ From reference (15).  ^g^ log P from reference (16). Calculated log D_6.5_ using log P and pK_a_.  ^h^ From reference (17)  ^I^ log D value measured at pH 7.4. From Reference (18). ADMET Predictor™ calculated values of 3.3 at pH 6.5.  ^j^ Experimental data measured at Uppsala University laboratories.  ^k^ From www.drugbank.ca  ^l^ log P from Reference (19). Calculated Log D_6.5_ using log P and pK_a_.  ^m^ From Reference (20).  ^n^ From Reference (21).  ^o^ From Reference (22).  ^p^ From Reference (23).  ^q^ From Reference (24)  ^r^ From Reference (25). |
| --- |

**Table S2.** Experimental Results Compared With Calculated Results from Model Compound Confirmation, Showing Effect of Buffer Capacity/Concentration and Effect of Bile Salts and Phospholipids

| **Effect of Buffer Capacity/Concentration** | | | | | |
| --- | --- | --- | --- | --- | --- |
| **Compound** | **Calculated Equivalent**  **Phosphate**  **Concentration (mM)** | **α (acids) or**  **β (bases) ^a^** | **Expected Effect Based Upon Recommendations?** | **Fold difference in dissolution rate ^b^** | |
|  |  |  |  | **With Bile Salts/ Phospholipids** | **Without Bile Salts/ Phospholipids** |
| Atenolol | 0.1 | 5.4 | No^c^ | Not tested | 1.3 |
| Dipyridamole | 0.2 | 11.9 | No | 1.0 | 1.0 |
| Ketoconazole | 0.2 | 12.6 | No | 1.8 | 0.9 |
| Tamoxifen | 0.3 | 12.0 | No | 1.1 | 1.0 |
| Flufenamic acid | 0.4 | 9.9 | Yes | 2.7 | 2.9 |
| Ibuprofen | 3.5 | 7.9 | Yes | 7.4 | 8.1 |
| Danazol ^d^ | - | – | No | 0.9 | 1.1 |
| Prednisolone ^d^ | – | – | No | 0.9 | 0.9 |
| **Effect of Bile Salts and Phospholipids (BS + PL)** | | | | | |
| **Compound** | **log D_6.5_ ^e^** | | **Expected Effect Based Upon Recommendations?** | **Fold difference in dissolution rate ^f^** | |
|  |  |  |  | **At 50 mM** | **At Equivalent** |
| Atenolol | -2.8 | | No | Not tested | Not tested |
| Dipyridamole | 3.6 | | Yes | 1.4 | 1.4 |
| Ketoconazole | 4.2 | | Yes | 0.7 | 1.5 |
| Tamoxifen | 4.5 | | Yes | 1.4 | 1.6 |
| Flufenamic acid | 3.7 | | Yes | 1.3 | 1.1 |
| Ibuprofen | 1.7 | | No | 1.1 | 1.0 |
| Danazol | 4.5 | | Yes | 1.7 | 1.7 |
| Prednisolone | 1.6 | | No | 1.0 | 1.0 |
| ^a^  α = pK_a_ – log S_o_ and β = pK_w_ – pK_a_ – log S_o_  ^b^ Fold difference in dissolution rate is the ratio of dissolution rate in 50 mM/equivalent buffer  ^c^ Although β < 11 and surface pH is calculated to increase, S_o_ is sufficiently high (Atenolol is a BCS 1 drug) such that surface pH increases to a high and similar extent in both 50 mM and 1 mM buffer. Therefore the difference in calculated dissolution rates is less than 2-fold.  ^d^ A 10 mM buffer was chosen as the low buffer concentration buffer for the experiments. An equivalent buffer cannot be calculated for these neutral compounds due to a lack of a relevant pKa.  ^e^ See Table S1 for references for log D_6.5_ values  ^f^ Fold difference in dissolution rate is the ratio of dissolution rate with BS + PL/blank buffer | | | | | |


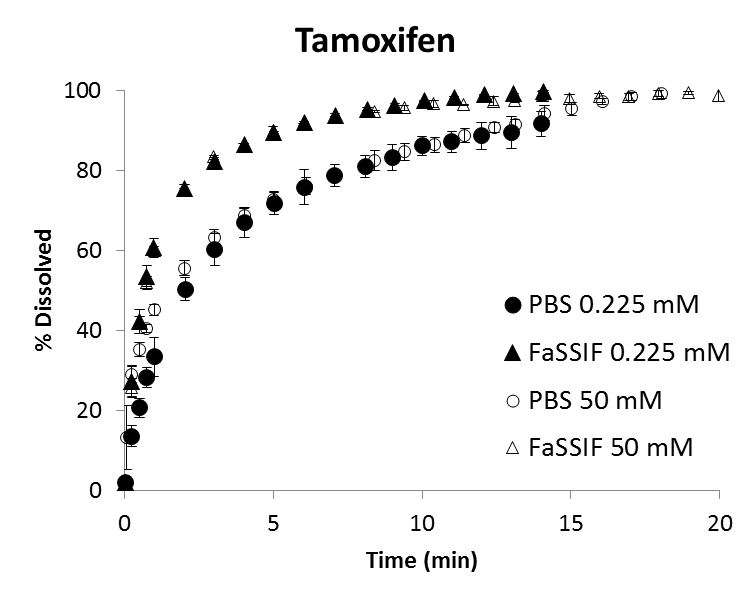

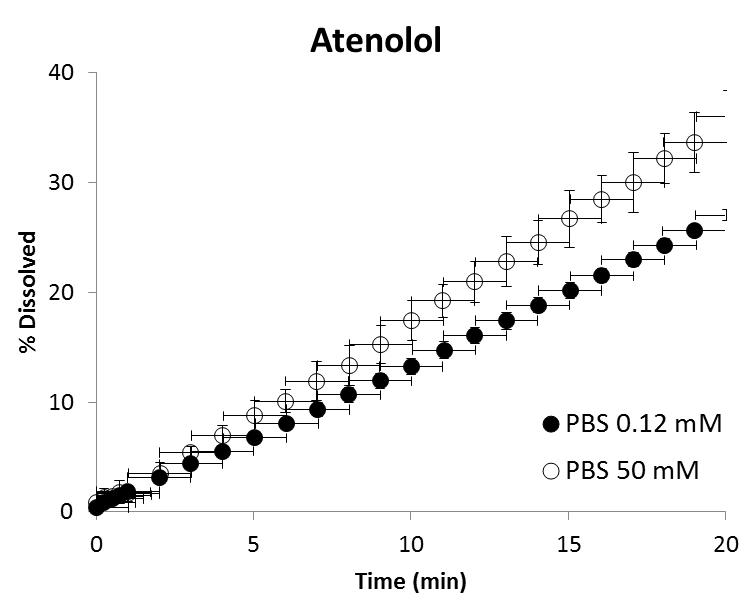

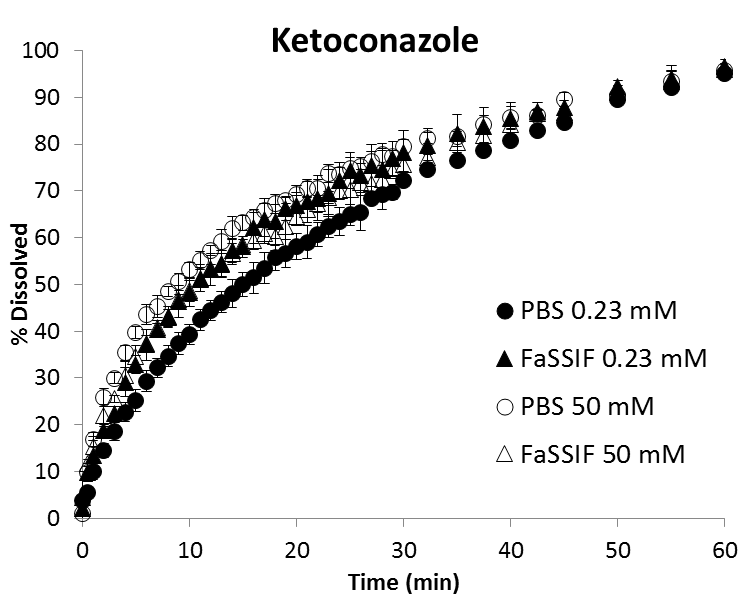
**
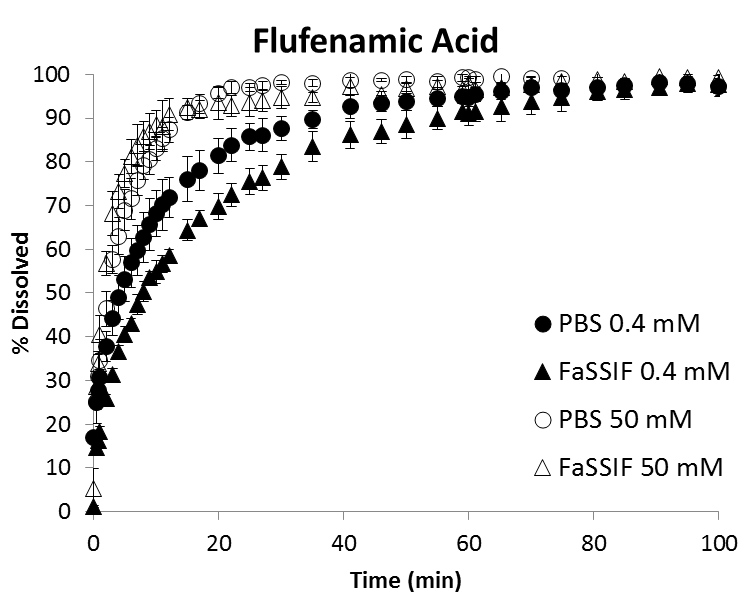
Figure S7. Dissolution Time Profiles for Model Weak Acids and Bases**


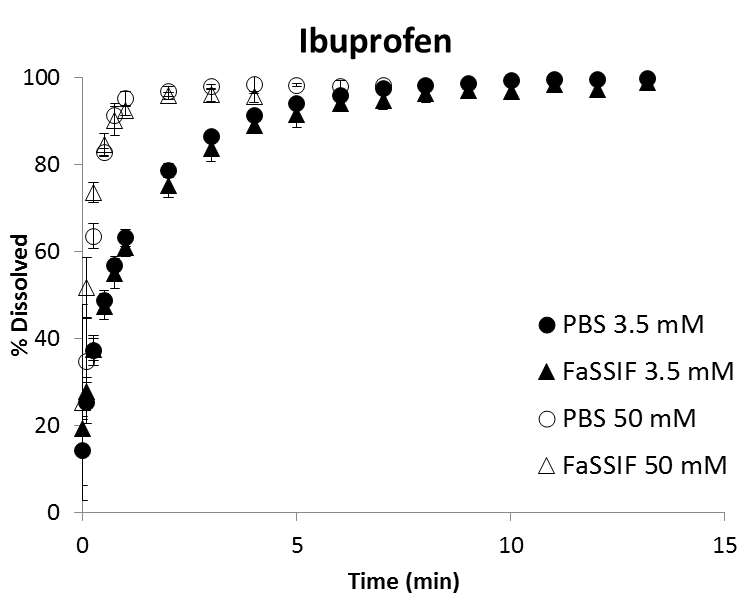

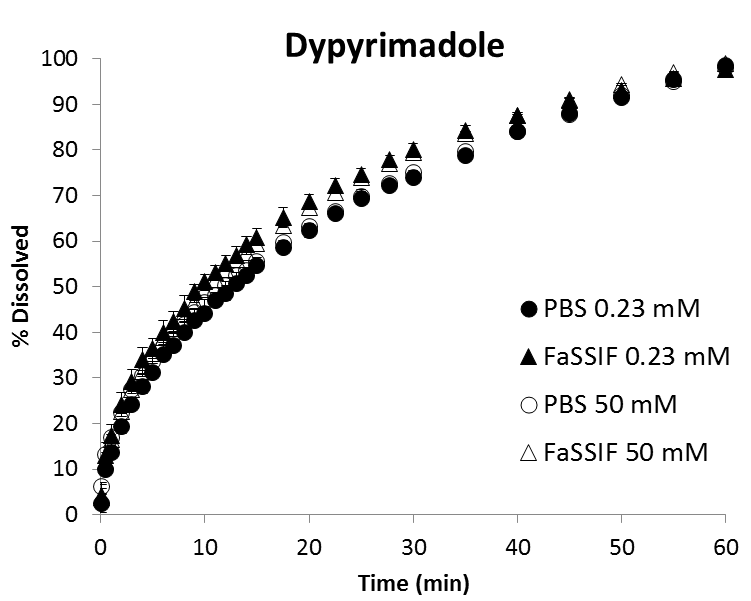


**
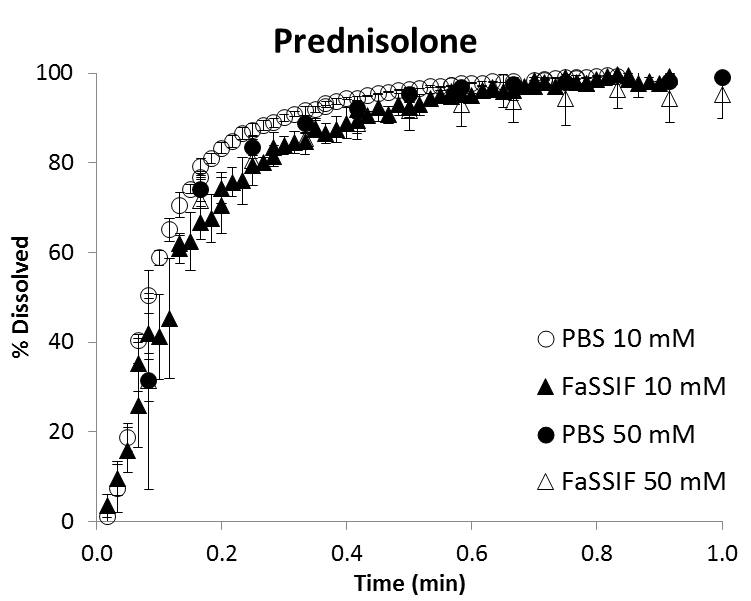

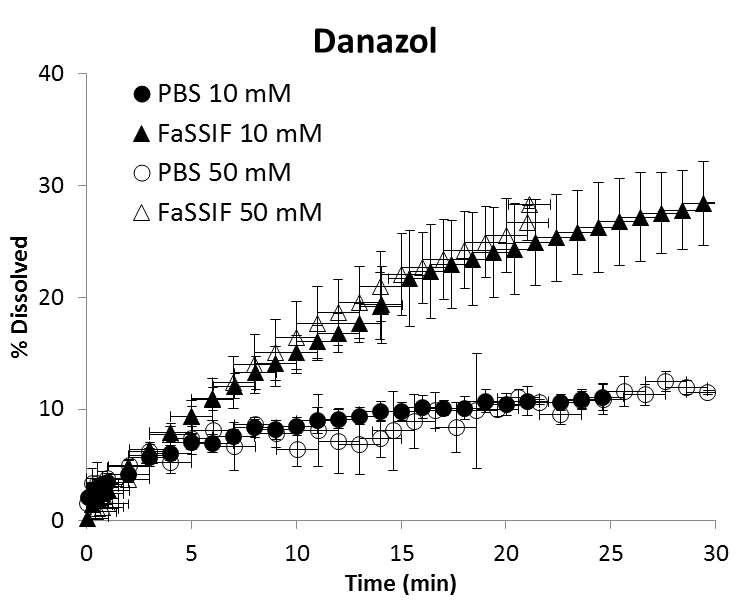
Figure S8. Dissolution Time Profiles for Model Neutral Drugs**

**References**

1. Sinko PJ, Martin AN. Martin's physical pharmacy and pharmaceutical sciences : physical chemical and biopharmaceutical principles in the pharmaceutical sciences. Philadelphia: Lippincott Williams & Wilkins; 2006.

2. Balakrishnan A, Rege BD, Amidon GL, Polli JE. Surfactant-mediated dissolution: Contributions of solubility enhancement and relatively low micelle diffusivity. Journal of Pharmaceutical Sciences. 2004;93(8):2064-75.

3. Okazaki A, Mano T, Sugano K. Theoretical dissolution model of poly-disperse drug particles in biorelevant media. Journal of Pharmaceutical Sciences. 2008;97(5):1843-52.

4. Amidon GE, Higuchi WI, Ho NFH. Theoretical and experimental studies of transport of micelle‐solubilized solutes. Journal of Pharmaceutical Sciences. 1982;71(1):77-84.

5. Sugano K, Okazaki A, Sugimoto S, Tavornvipas S, Omura A, Mano T. Solubility and dissolution profile assessment in drug discovery. Drug Metab Pharmacokinet. 2007;22(4):225-54.

6. Hayduk W, Laudie H. Prediction of diffusion coefficients for nonelectrolytes in dilute aqueous solutions. AIChE Journal. 1974;20(3):611-5.

7. Shekunov B. Theoretical Analysis of Drug Dissolution in Micellar Media. Journal of Pharmaceutical Sciences. 2017;106(1):248-57.

8. Andersson SBE, Alvebratt C, Bergstrom CAS. Rapid determination of dissolution-limited drug absorption and effect of particle size reduction. Pharm Res. Submitted in 2017.

9. Andersson SBE, Alvebratt C, Bevernage J, Bonneau D, da Costa Mathews C, Dattani R, et al. Interlaboratory Validation of Small-Scale Solubility and Dissolution Measurements of Poorly Water-Soluble Drugs. Journal of pharmaceutical sciences. 2016;105(9):2864-72.

10. Fagerberg JH, Bergstrom CAS. Intestinal solubility and absorption of poorly water soluble compounds: predictions, challenges and solutions. Therapeutic delivery. 2015;6(8):935-59.

11. Avdeef A, Berger CM, Brownell C. pH-metric solubility. 2: correlation between the acid-base titration and the saturation shake-flask solubility-pH methods. Pharm Res. 2000;17(1):85-9.

12. Mohsen-Nia M, Ebrahimabadi AH, Niknahad B. Partition coefficient n-octanol/water of propranolol and atenolol at different temperatures: Experimental and theoretical studies. The Journal of Chemical Thermodynamics. 2012;54:393-7.

13. Marmol AL, Box K, Wagner KG. Mechanistic Evaluation of Supersaturation and Solubility Enhancement for "Chasing" and "Non Chasing" APIs using Colloids.

14. Box K. In: personal communication to Mudie DM, editor. Sirius Analytical, Ltd.2017.

15. Hsieh YL, Ilevbare GA, Van Eerdenbrugh B, Box KJ, Sanchez-Felix MV, Taylor LS. pH-Induced precipitation behavior of weakly basic compounds: determination of extent and duration of supersaturation using potentiometric titration and correlation to solid state properties. Pharm Res. 2012;29(10):2738-53.

16. Jacobs GA, Gerber M, Malan MM, du Preez JL, Fox LT, du Plessis J. Topical delivery of acyclovir and ketoconazole. Drug Deliv. 2016;23(2):631-41.

17. Sun N, Avdeef A. Biorelevant pK(a) (37 degrees C) predicted from the 2D structure of the molecule and its pK(a) at 25 degrees C. J Pharm Biomed Anal. 2011;56(2):173-82.

18. Jena SK, Singh C, Dora CP, Suresh S. Development of tamoxifen-phospholipid complex: Novel approach for improving solubility and bioavailability. International Journal of Pharmaceutics. 2014;473(1–2):1-9.

19. Hadgraft J, du Plessis J, Goosen C. The selection of non-steroidal anti-inflammatory agents for dermal delivery. Int J Pharm. 2000;207(1-2):31-7.

20. Palmer DS, Llinas A, Morao I, Day GM, Goodman JM, Glen RC, et al. Predicting intrinsic aqueous solubility by a thermodynamic cycle. Mol Pharm. 2008;5(2):266-79.

21. Krieg BJ, Taghavi SM, Amidon GL, Amidon GE. In Vivo Predictive Dissolution: Comparing the Effect of Bicarbonate and Phosphate Buffer on the Dissolution of Weak Acids and Weak Bases. Journal of Pharmaceutical Sciences. 2015;104(9):2894-904.

22. Mudie DM, Shi Y, Ping H, Gao P, Amidon GL, Amidon GE. Mechanistic analysis of solute transport in an in vitro physiological two-phase dissolution apparatus. Biopharmaceutics & Drug Disposition. 2012;33(7):378-402.

23. Mithani SD, Bakatselou V, TenHoor CN, Dressman JB. Estimation of the increase in solubility of drugs as a function of bile salt concentration. Pharm Res. 1996;13(1):163-7.

24. Soderlind E, Karlsson E, Carlsson A, Kong R, Lenz A, Lindborg S, et al. Simulating Fasted Human Intestinal Fluids: Understanding the Roles of Lecithin and Bile Acids. Molecular Pharmaceutics. 2010;7(5):1498-507.

25. Machatha SG, Yalkowsky SH. Comparison of the octanol/water partition coefficients calculated by ClogP, ACDlogP and KowWin to experimentally determined values. Int J Pharm. 2005;294(1-2):185-92.
